# Bax Activation Initiates the Assembly of a Multimeric Catalyst that Facilitates Bax Pore Formation in Mitochondrial Outer Membranes

**DOI:** 10.1371/journal.pbio.1001394

**Published:** 2012-09-25

**Authors:** Yulia Kushnareva, Alexander Y. Andreyev, Tomomi Kuwana, Donald D. Newmeyer

**Affiliations:** 1 La Jolla Institute for Allergy and Immunology, La Jolla, California, United States of America; 2 Department of Pharmacology, University of California San Diego, La Jolla, California, United States of America; John Hopkins University School of Medicine, Baltimore, United States of America

## Abstract

Bax/Bak-mediated mitochondrial outer membrane permeabilization (MOMP) is essential for “intrinsic” apoptotic cell death. Published studies used synthetic liposomes to reveal an intrinsic pore-forming activity of Bax, but it is unclear how other mitochondrial outer membrane (MOM) proteins might facilitate this function. We carefully analyzed the kinetics of Bax-mediated pore formation in isolated MOMs, with some unexpected results. Native MOMs were more sensitive than liposomes to added Bax, and MOMs displayed a lag phase not observed with liposomes. Heat-labile MOM proteins were required for this enhanced response. A two-tiered mathematical model closely fit the kinetic data: first, Bax activation promotes the assembly of a multimeric complex, which then catalyzes the second reaction, Bax-dependent pore formation. Bax insertion occurred immediately upon Bax addition, prior to the end of the lag phase. Permeabilization kinetics were affected in a reciprocal manner by [cBid] and [Bax], confirming the “hit-and-run” hypothesis of cBid-induced direct Bax activation. Surprisingly, MOMP rate constants were linearly related to [Bax], implying that Bax acts non-cooperatively. Thus, the oligomeric catalyst is distinct from Bax. Moreover, contrary to common assumption, pore formation kinetics depend on Bax monomers, not oligomers. Catalyst formation exhibited a sharp transition in activation energy at ∼28°C, suggesting a role for membrane lipid packing. Furthermore, catalyst formation was strongly inhibited by chemical antagonists of the yeast mitochondrial fission protein, Dnm1. However, the mammalian ortholog, Drp1, was undetectable in mitochondrial outer membranes. Moreover, ATP and GTP were dispensable for MOMP. Thus, the data argue that oligomerization of a catalyst protein, distinct from Bax and Drp1, facilitates MOMP, possibly through a membrane-remodeling event.

## Abstract 2

Bax promotes mitochondrial permeabilization during apoptosis via a phase-transition-like event in the membrane and oligomerization of a catalyst molecule that facilitates Bax pore formation.

## Author Summary

Mitochondria are the key energy-producing structures inside cells, but are also crucial players in a common form of programmed cell death, apoptosis. A critical event in mitochondrion-driven apoptosis involves the formation of large pores in the mitochondrial outer membrane (MOM). These pores cause long-term damage to mitochondria and also allow mitochondrial proteins to escape and accelerate cell death. Previous studies have revealed that the protein Bax when activated can form pores in protein-free membranes and that it, along with Bak, is involved in the formation of mitochondrial pores, but the process remains unclear. We now show, however, that in naturally derived MOMs, Bax is assisted by another resident MOM protein, which we term the “catalyst,” and whose identity is still unknown. The mechanism involves two distinct stages. First, activated Bax activates the catalyst protein, causing multiple catalyst molecules to assemble into a larger structure (a complex). In the second stage, this catalyst complex in turn facilitates Bax-driven pore formation. Our data also reveal some unexpected details of the pore formation process; in particular, it appears that catalyst activation involves a physical change in the molecular arrangement of the membrane. Furthermore, contrary to what was previously assumed, pore formation does not require Bax molecules themselves to assemble together into larger complexes.

## Introduction

Mitochondria are well known to be essential for cell life, as they produce ATP and other products of key biosynthetic pathways. Intriguingly, mitochondria are also often critical for cell death [1]–[4]. In vertebrates, apoptotic cell death typically involves a canonical “intrinsic” apoptotic pathway that depends on mitochondrial outer membrane permeabilization (MOMP). MOMP is induced by the pro-apoptotic Bcl-2 family proteins Bax and/or Bak [5]–[12].

During MOMP, supramolecular pores are formed that are permeable even to large proteins. These pores lead to cell death in two ways: first, they allow proteins normally residing in the mitochondrial intermembrane space to be released into the cytoplasm, where these proteins then activate or enhance caspase-dependent death pathways. In particular, cytochrome c and Smac/DIABLO promote Apaf-1-dependent activation of Caspase-9 and the “executioner” caspases-3, -6, and -7, leading to apoptosis [13]–[18]. Second, even when this Apaf-1-dependent pathway is blocked, outer membrane pores lead to cell death by initiating a slow but progressive loss of mitochondrial function. As a result, cellular energy stockpiles become depleted and DNA replication slows to a halt, within two cell division cycles [19]. Normally, this disruption of mitochondrial bioenergetic function leads to an absolute loss of clonogenic survival. However, when the protein GAPDH is overexpressed and caspases are inhibited, some cells manage to survive and proliferate. To do so, they must first maintain energy by boosting glycolysis and autophagy. Then, they must restore the mitochondrial network by biogenesis, starting from a small remnant of mitochondria that evade MOMP [4],[20].

The precise molecular mechanism of MOMP is not yet understood. Earlier, we showed that the process of Bax/Bak-dependent MOMP that occurs in apoptotic cells could be reproduced in vitro using resealed dextran-loaded mitochondrial outer membrane vesicles (OMVs) [9],[21]. This demonstrates that the core MOMP machinery is intrinsic to the outer membrane and does not require components from the intermembrane space, the inner membrane, or the mitochondrial matrix. (Of course, the ability of isolated OMVs to undergo MOMP does not imply that interior mitochondrial structures are irrelevant to apoptosis. For example, crista junctions can influence cell fate by controlling the retention of proteins such as cytochrome c and Smac within cristae [22]–[25].) Previous studies from others and ourselves have modeled Bax-dependent membrane pore formation with simple in vitro systems consisting of protein-free liposomes mixed with recombinant Bax and “direct activator” BH3-only proteins—for example, cBid and BimS (e.g., [9],[21],[26],[27]). However, it has been unclear whether liposome systems accurately reflect the physiological events in mitochondria.

Here, to help elucidate the mechanisms of MOMP in native mitochondrial membranes, we undertook a thorough biochemical kinetic analysis of Bax-dependent MOMP using isolated outer membranes. Our data show that the native membranes display a more sensitive and kinetically more complex response to Bax than that observed with liposomes. Heat-labile MOM proteins are required for this enhanced response. Thus, although liposome systems have revealed an intrinsic pore-forming function of Bax, they do not fully reproduce the permeabilization process in apoptotic mitochondria.

Our kinetic studies confirmed the existence of an outer membrane-resident “receptor” for cBid [28],[29]. Moreover, our studies revealed a reciprocal relationship between the effective concentrations of cBid and Bax. This is evidence for a transient collision interaction between cBid and Bax and is clear experimental support for the “hit-and-run” hypothesis of cBid-induced direct Bax activation.

Importantly, we developed a mathematical model to explain the observed kinetics of OMV permeabilization. This, in combination with biochemical studies, revealed some unexpected aspects of Bax-dependent MOMP. In particular, we showed that, during the lag phase, activated Bax triggers the multimerization of a catalyst molecule that facilitates another Bax-dependent event, the formation of large membrane pores. However, our data show that the catalyst is distinct from Bax, and contrary to what is often assumed, Bax oligomerization is unrelated to the kinetics of MOMP. Furthermore, Bax insertion and membrane recruitment were temporally early events, beginning just after the addition of Bax.

The catalyst assembly reaction displayed phase transition-like behavior, raising the possibility that it involves a membrane-remodeling event. Based on recent studies [30],[31], a prime candidate for the catalyst was the fission-related protein Drp1. Considering this, we tested the effect of compounds (analogs of mdivi-1) that were originally identified as mitochondrial fission inhibitors in yeast. These compounds inhibit the GTPase activity of Dnm1, the yeast ortholog of Drp1. Although the active mdivi-1 analogs do not affect recombinant Drp1's GTPase activity, they do inhibit mitochondrial fission in mammalian cells and MOMP in isolated vertebrate mitochondria [31]. Strikingly, we found that mdivi-1 analogs inhibited the formation of the MOMP-related catalyst. However, because Drp1 was undetectable in mitochondrial outer membranes, and because GTP and ATP were not required for pore formation, our data argue that a MOM-resident protein distinct from Drp1 participates in catalyst assembly and serves as a target of chemical Dnm1 inhibitors. We propose that this catalyst activity promotes pore formation, by facilitating the redistribution of Bax in the MOM or by directly enhancing the pore-formation function of Bax.

## Results and Conclusions

**Figure 1:**
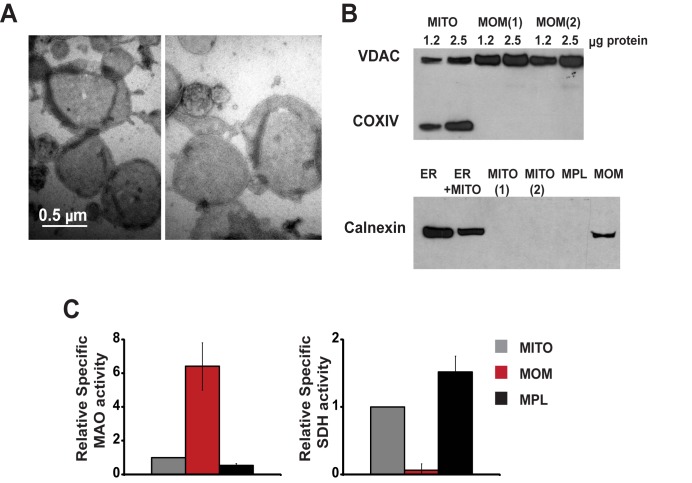
Mitochondrial outer membranes isolated from rat liver form vesicles that are essentially devoid of the mitochondrial inner membrane. Negatively stained outer membrane vesicles (OMVs) were visualized by electron microscopy (A). The purity of OMVs was evaluated by Western blot analysis (B) and by measurements of enzyme activities (C) of marker proteins: VDAC and monoaminooxidase (MAO) for the mitochondrial outer membrane (MOM); cytochrome c oxidase subunit (COXIV) and succinate dehydrogenase (SDH) for the inner membrane; and calnexin for the ER. Shown in (B) are mitochondria (MITO) and two preparations of MOM (upper panel); ER and light mitochondrial fraction (ER+MITO) in comparison with two preparations of mitochondria, mitoplasts (MPL), and MOM (lower panel). Samples were loaded at 2.5 µg of protein or as indicated. (C) Specific SDH and MAO activities in MOM and mitoplasts relative to the corresponding activities in whole mitochondria. Activities were measured in four to six preparations and data are presented as means ± S.E.

To uncover features of Bax-dependent pore formation in a near-physiological but experimentally tractable system, we used isolated mitochondrial outer membranes (MOMs). Earlier we showed that isolated MOMs from Xenopus laevis eggs spontaneously reseal to form “Outer Membrane Vesicles” (OMVs) that can entrap fluorescent dextrans [9]. Xenopus OMVs become permeabilized upon the addition of recombinant BH3-only proteins such as cleaved Bid (cBid), and this permeabilization is inhibited by the anti-apoptotic Bcl-xL protein. Here, we prepared OMVs from rat liver mitochondria (Figure 1A), which were advantageous for our studies because they do not respond to added cBid alone (as shown by others; [32],[33]), but become permeabilized by a combination of cBid and Bax. Immunoblot analysis showed that rat liver OMVs lack detectable amounts of Bax ([32],[34] and Figure S1C). They contain some Bak (albeit less than the amount in mouse liver mitochondria; not shown), but this resident Bak protein is apparently insufficient to permeabilize mitochondria, or is perhaps inhibited by another MOM protein such as VDAC2 [35]. Therefore, we could measure the quantitative response to recombinant Bax in the absence of endogenous Bax-like activity. Purified rat liver OMVs were largely devoid of inner mitochondrial membrane proteins, as shown by immunoblots (Figure 1B). Contamination with the inner membrane was ≈5% based on succinate dehydrogenase activity (Figure 1C). As expected, OMVs were highly enriched in the outer membrane proteins VDAC and monoaminooxidase (Figure 1B,C). We also detected the ER protein Calnexin in OMV preparations (Figure 1B), consistent with the physical association of mitochondria and ER [36].

### Kinetics of MOMP in Native Membranes versus Liposomes

**Figure 2:**
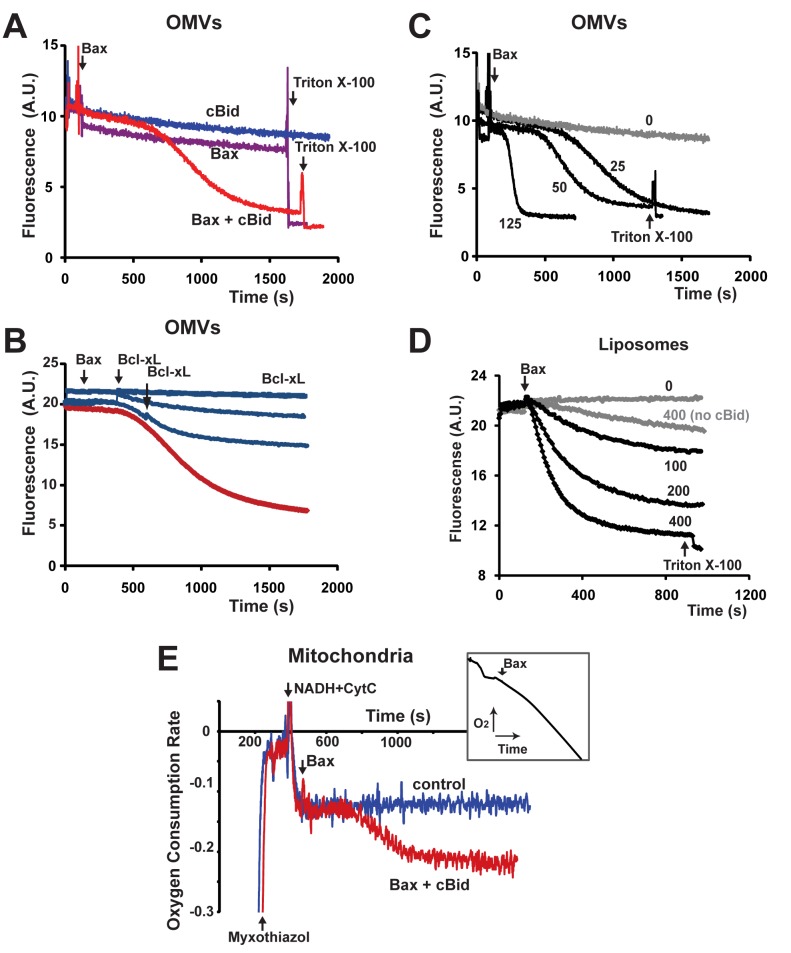
cBid/Bax–dependent membrane permeabilization: native mitochondrial outer membranes and protein-free liposomes display different permeabilization kinetics, and native membranes respond to lower concentrations of Bax. Dextran release was measured continuously in rat liver OMVs (A–C) and defined liposomes (D). OMVs were loaded with either 10 kDa dextran-cascade blue (A,C) or 70 kDa dextran-fluorescein (B). Bax concentration was 25 nM (A,B) or as indicated (C,D). Arrows indicate additions of Bax and 0.05% Triton X-100. When present, cBid (40 nM) was added 2–3 min prior to Bax. Bcl-xL (1 µM) was added 2 min before Bax or at later points indicated by arrows (B; blue lines). (E) Respiration-based continuous measurements of Bax-induced MOMP in isolated rat liver mitochondria. Mitochondria were incubated in a KCl-based respiration buffer containing 0.3 µM FCCP, 2 µM myxothiazol (Myxo), 2 mM NADH, and 80 µM cytochrome c (CytC). Under these conditions, the rate of NADH-dependent respiration depends on the rate of cytochrome c permeation through the MOM and therefore reflects the kinetics of MOMP. Arrows indicate additions of myxothiazol, NADH, CytC, and Bax (60 nM) mixed with cBid (40 nM). Control trace (blue line): no cBid/Bax added. Data are plotted as the first derivative of oxygen concentration (i.e., respiration rate) versus time. The insert shows the original oxygen consumption curve, in the presence of cBid and Bax. Note that the kinetics of MOMP in whole mitochondria and OMVs are similarly biphasic. Data shown are representative of at least three independent experiments. See also Figure S1.

To study the kinetics of MOMP, we continuously measured the release of OMV-entrapped fluorescent dextrans (either Cascade Blue- or fluorescein-labeled), using specific antibodies that are excluded from intact OMVs but quench the fluorescence of released fluorophores. This quenching was rapid, as evidenced by the near-instantaneous drop in the fluorescence signal upon membrane permeabilization by Triton X-100 (Figure 2A). Rat liver OMVs, like whole rat liver mitochondria, responded to neither cBid nor Bax proteins alone, even if cBid was added at high concentrations (Figure S1A). As expected, OMVs became permeabilized by cBid and Bax added together (Figure 2A), and the anti-apoptotic Bcl-xL protein abrogated this response (Figure 2B). Consistent with earlier results, dextran release from Xenopus OMVs was induced by cBid alone and more potently by mixtures of cBid and Bax (Figure S1B). Interestingly, both in rat liver and Xenopus OMVs, the kinetics were biphasic, with a pronounced early lag phase. Bax mixed with either recombinant BimS protein (Figure S6C), or Bim BH3-domain peptide (unpublished data) produced similar biphasic kinetics. These complex MOMP kinetics were strikingly different from the monophasic kinetics of cBid/Bax-induced dextran release from protein-free liposomes (Figure 2D). Both synthetic liposomes (Figure 2D) and liposomes formed from extracted mitochondrial phospholipids (Figure S1D) displayed monophasic permeabilization kinetics, similar to ones observed in other studies [27],[37]–[39]. Moreover, OMVs, despite showing an initial lag, were substantially more responsive than liposomes to lower concentrations of Bax (Figure 2C,D and Figure S1). Thus, liposomes only partially reproduce the process of Bax-induced pore formation in native membranes.

To determine whether the kinetics of dextran release from OMVs faithfully reflect those of MOMP in mitochondria, we developed a continuous kinetic assay for whole mitochondria that measures the ability of exogenous reduced cytochrome c to traverse the outer membrane [40],[41]. Here, normal mitochondrial respiration was blocked by myxothiazol (a Complex III inhibitor) to prevent interference with MOMP assessment. Complex IV-dependent respiration driven by oxidation of external NADH was sustained by the continuous reduction of exogenous cytochrome c via the flavoprotein (Fp_5_)-cytochrome b5 reductase complex [42],[43]. Under control conditions, the rate of respiration was very low because exogenous cytochrome c cannot pass through the intact outer mitochondrial membrane. However, the addition of cBid and Bax increased the respiration rate, after a lag phase, and Bcl-xL inhibited this response (Figures 2E and S1E). Thus, whole mitochondria and OMVs displayed very similar biphasic kinetics of cBid/Bax-induced permeabilization.

From these studies, we conclude that the native MOM has a mechanism that potentiates Bax-mediated pore formation, after a certain lag. We considered the possibility that this lag was caused by a requirement for the accumulation or maturation of a factor within the membranes. However, we found that preincubation of the membranes (with or without cBid) prior to the addition of Bax did not eliminate the lag phase (unpublished data). In other words, the lag phase does not represent a constitutive or cBid-induced process in the MOM, but is initiated by Bax (upon activation).

Another possible explanation for the lag phase could have been a time-dependent de-repression of Bax inhibition by endogenous anti-apoptotic Bcl-2-family proteins. To test this possibility, we preincubated OMVs with two BH3 peptides (Bad and Noxa) that together inhibit most of the anti-apoptotic proteins [21] or with ABT-737, a chemical inhibitor of several anti-apoptotic proteins, including Bcl-xL [44]. The peptides only slightly accelerated cBid/Bax-induced dextran release (Figure S2A), possibly reflecting the weak ability of Noxa to activate Bax directly [45]. ABT-737 also had no effect unless recombinant Bcl-xL was added to inhibit MOMP (Figure S2B). These data indicate that endogenous anti-apoptotic Bcl-2-family proteins are not highly active in rat liver OMVs. An additional experiment performed with liposomes showed that when cBid/Bax-induced permeabilization was partially inhibited by Bcl-xL, the subsequent addition of an increased amount of cBid reversed the inhibition almost immediately (Figure S2C). Thus, displacement interactions among Bcl-2-family proteins are rapid and unlikely to explain the lag phase.

**Figure 3:**
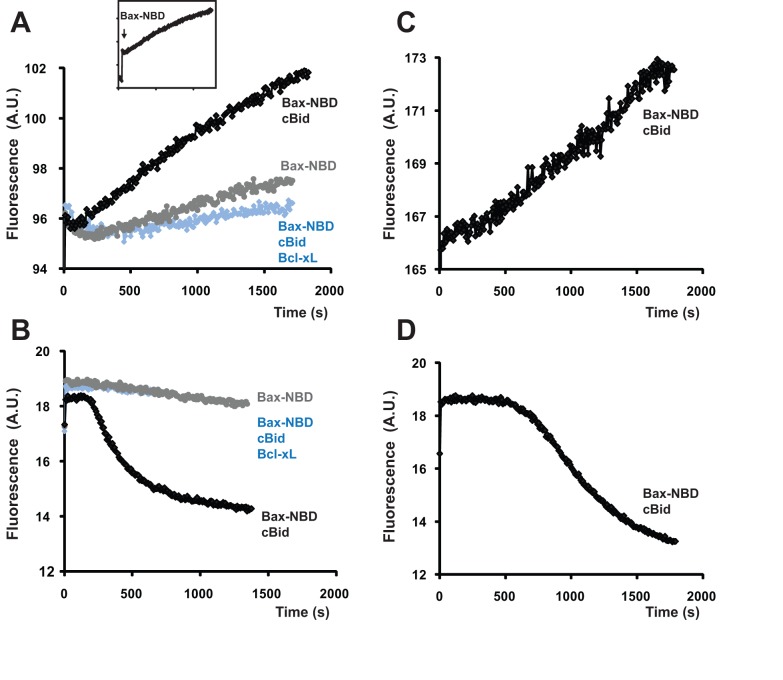
In OMVs, Bax membrane integration begins immediately upon addition of Bax, while dextran release displays a significant lag-phase. Bax membrane integration was measured by an increase in NBD-labeled Bax fluorescence. Kinetics of Bax insertion (A,C) and dextran-Cascade Blue release (B,D) were measured at two Bax-NBD concentrations: 100 nM (A,B) and 40 nM (C,D). Bax-NBD was added at time zero. When present, cBid (40 nM) and Bcl-xL (2 µM) were added 2–3 min prior to Bax-NBD. The insert shows an initial instantaneous increase in fluorescence upon Bax-NBD addition, followed by a gradual increase in the NBD signal. This time-dependent change in NBD fluorescence (reflecting membrane insertion) is shown on the graph (A). Bax membrane integration and dextran release both occur in a cBid-dependent, Bcl-xL-inhibitable manner but display different kinetics (see also Figure S3 showing measurements of Bax membrane binding by an independent method). Thus, Bax integration is rapid and cannot be the rate-limiting step occurring during the lag phase. See also Figure S2, showing that displacement reactions of Bcl-2-family proteins are also rapid and cannot explain the lag phase.

In liposomes, Bax integration into the membrane is relatively slow compared with tBid-Bax interaction and pore formation and thus apparently rate-limiting [26]. Because permeabilization kinetics were more complex in native mitochondrial membranes, we asked whether Bax integration was similarly rate-limiting in OMVs, or whether another step was responsible for the lag phase. For continuous measurements of Bax insertion in OMVs, we adapted a fluorometric method [26] that takes advantage of the increased fluorescence of Bax labeled with NBD [N,N′-dimethyl-N-(iodoacetyl)-N′-(7-nitrobenz-2-oxa-1,3-diazol-4yl)ethylenediamine] that occurs upon membrane insertion. We could thus compare the kinetics of Bax integration (increase in NBD fluorescence) and MOMP (loss of dextran-Cascade Blue fluorescence). As shown in Figure 3, Bax began to integrate into the membrane almost immediately upon its addition in the presence of cBid, whereas dextran release began only after a 2–8 min lag. As expected, both the fluorescence increase and dextran release induced by Bax-NBD were prevented by Bcl-xL by cBid omission (Figure 3A,B). To examine the kinetics of Bax association with membranes in another way, we re-isolated OMVs at various times after Bax addition, using a density gradient float-up method, and then measured Bax levels by immunoblotting [21]. The results show that cBid recruits Bax to OMVs well before the lag phase is completed (Figure S3). Thus, two independent methods demonstrate that Bax is recruited to outer membranes very early in the MOMP process. We conclude that the lag phase reflects another time-dependent event occurring in the native MOM, after Bax membrane insertion. We considered the possibility that this second event was merely the accumulation of a global threshold amount of integrated Bax. However, if that were true, we would expect a similar global buildup of Bax to be required in liposomes. But as liposomes exhibited no delay in pore formation following Bax insertion, that explanation is unlikely.

### Kinetic Model of Bax-Induced Pore Formation

To help explain the mechanism of MOMP, we developed a biochemical reaction model that fit the observed kinetic data (see Materials and Methods). To enable this analysis we made the simplifying assumption that each vesicle releases all of its dextran content at once. (Flow cytometric analysis of individual OMVs revealed that OMVs do display this mode of near-instantaneous dextran release; Kuwana et al., unpublished). Importantly, this assumption implies that the normalized fluorescence of the OMV suspension, corresponding to the fraction of dextran molecules that remain entrapped in vesicles, also equals the fraction of intact vesicles (which is what we model mathematically). Also implicit in this assumption is the idea that the formation of the first pore in a given OMV is sufficient to allow complete dextran release; this is consistent with observations made by cryo-electron microscopy that activated Bax can cause the formation of large openings in liposomes [28]. Importantly, we observed that dextrans of widely different sizes showed similar release kinetics (Figure S4). As we assumed that the vesicles all behave in a binary fashion (either intact or permeabilized), it followed that, for the purposes of mathematical modeling, we could regard the OMVs to be mathematically equivalent to molecular entities, which also exist in binary states (reacted or unreacted). We could therefore use a traditional enzyme kinetics approach to model the vesicle population.

Although the vesicles individually behaved as binary entities, as an ensemble they became permeabilized in a non-synchronous (quasi-stochastic) manner, yielding an exponential-like dextran-release curve following the lag phase. Consistent with this, we found that Bcl-xL added at various times during the rapid phase prevented almost all subsequent dextran release (Figure 2B). In other words, Bcl-xL preserved the integrity of most of the vesicles that had yet to be permeabilized.

**Figure 4:**
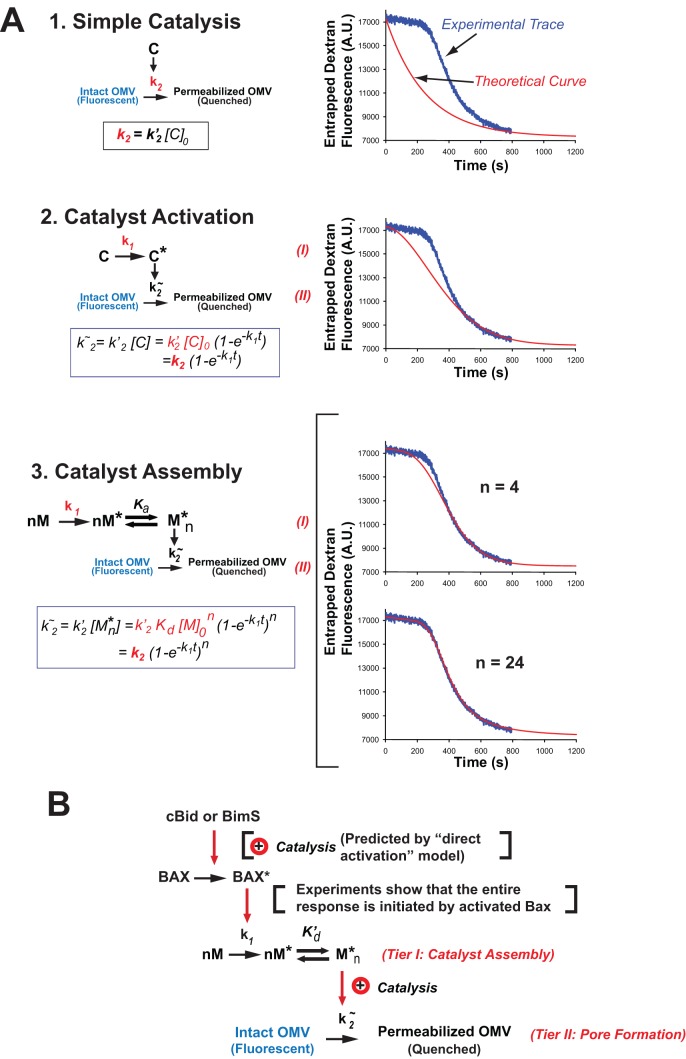
Development of the two-reaction kinetic model of MOMP describing dextran release curves. (A). A two-tiered model is required to fit the experimental data. Models of increasing complexity are shown on the left. In these models, k_1_ is a first-order kinetic constant; k_2^∼^_ is a pseudo-first-order constant dependent on the model (the expression for k_2^∼^_ is shown in the box in each panel). *C* and C^*^ denote inactive and active forms of a monomeric pore-forming catalyst. *M*, M^*^, and M^*^_n_ are inactive and activated monomers and an activated n-mer of a multimeric catalyst, respectively. The last two models differ only with respect to the degree of multimerization n (*n* = 4 or *n* = 24). k_2_ is a combination constant that can be determined, along with k_1_, from a curve-fitting procedure (illustrated by graphs on the right). Depending on the model, k_2_ may include initial concentrations of catalyst ([C]_0_) or of the monomer ([M]_0_), the association constant (K_a_) of the multimeric complex M^*^_n_ and the degree of multimerization (n). In the curve-fitting graphs, the blue line is a representative experimental curve. Red lines are theoretical curves corresponding to each of the four models. Derivation of the expression for k_2^∼^_ for the “catalyst assembly” model is described in detail in the Materials and Methods section. Curve fitting was performed using GraphPad Prizm. The parameter n was constrained to 24. Parameters of the resulting fit were as follows: Fm = 7,191±42, Fo = 10,089±42, k1 = 0.00869±0.00004, k2 = 0.00343±0.00005, k3 = 0.00012±0.00001; data are mean ± SE. Statistical characteristics of the fit were as follows: degrees of freedom = 787, *R* ^2^ = 0.9987, absolute sum of squares = 14,440,000, Sy.x = 135.5. As evident from a very small standard error, narrow confidence intervals (unpublished data) and high *R* ^2^, the model of a multimeric complex with high degree of multimerization (Figure 4A, panel 3) produces an exceptionally tight fit. The “Stable catalyst” model describes a classic exponential decay process. Adding a requirement for catalyst activation leads to a sigmoid curve that reflects increasing concentration of the catalyst over time; however, it does not produce a prolonged lag phase. A prolonged lag phase is observed for the model of “Catalyst Assembly” due to the introduction of a multimerization step that makes the process highly cooperative and requires a “threshold” amount of activated monomer to be accumulated before the active catalyst M^*^_n_ can be formed, promoting vesicle permeabilization. Increasing the degree of multimerization (n) required for the formation of the active catalytic complex sharpens the transition between the lag and active phases. See also Figure S4, an experiment supporting the key assumption that dextran release is all-or-nothing. (B) Molecular scheme, combining the kinetic model in (A) with the “hit and run” concept for Bax activation drawn from the literature as well as experimental observations showing that the kinetic curves are initiated by activated Bax (Bax mixed with direct activator BH3-only proteins or BH3 peptide). See text for further explanation.

To explain the observed kinetics, we explored various basic reaction schemes (Figure 4A). The simplest of these did not match the data, but a somewhat more complex reaction scheme yielded essentially a perfect curve-fit. This scheme involves two coupled reactions, where the first tier (reaction I) generates a catalyst for the second tier (reaction II; Figure 4 and Materials and Methods). In reaction I, the catalyst is assembled from monomer subunits (M) that must first be activated (to M*) and then undergo multimerization (to M*_n_). Reaction II is simply the conversion of an intact vesicle to a permeabilized vesicle, via the formation of a supramolecular pore.

For each set of experimental conditions, we obtained a curve-fit of the equation in Figure 4A (“Catalyst Assembly”) to the time-course of continuous fluorometric measurements. This yielded values of two kinetic constants: k_1_ for the rate of catalyst assembly, which essentially characterizes the duration of the initial lag phase, and k_2_ for the rate of pore formation, which corresponds to the maximum slope of the rapid kinetic phase. The third variable parameter in the curve-fits is *n*, which corresponds to the average number of subunits assembled in the catalyst complex and is manifested in the sharpness of the transition between the two kinetic phases. Note that as *n* approaches 1, the “Catalyst Assembly” model collapses into “Catalyst Activation.” In general, we found that for reasonably good curve-fits, *n* had to be at least ∼12. However, the theoretical curves were not especially sensitive to changes in *n* at such magnitudes, and therefore, we could not measure this parameter precisely. Figure 4A (bottom) shows examples with *n* = 4 (a poor fit) and *n* = 24 (essentially a perfect fit to the experimental data). Figure 4B shows how this kinetic model can be accommodated into a molecular scheme by incorporating our data showing that the entire kinetic response, including the lag phase, was initiated by cBid or BimS-induced Bax activation, which is widely hypothesized to occur through a “hit-and-run” mechanism.

### Mechanistic Roles of Bax and cBid in Pore Formation

**Figure 5:**
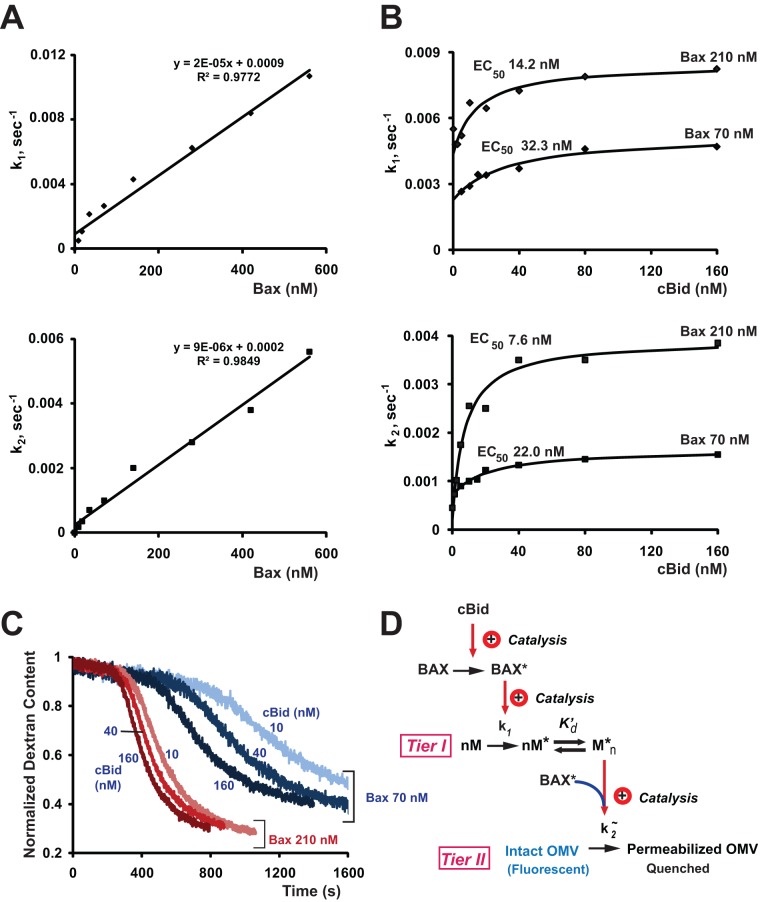
The kinetic parameters showed saturation with respect to cBid, but not Bax concentrations, and exhibited an inverse relationship between [cBid] and [Bax]. (A) Dependence of the rate constants on Bax concentration was linear. Some small deviations from linearity at low Bax concentrations probably reflect systematic error due to the need for long incubations under those conditions. (B) Dependence of the rate constants on cBid concentration was saturable and displayed an inverse relationship with the amount of Bax (lower EC_50_ values at higher Bax concentration). The assay was performed as in Figure 2A,C. Data shown in (A) and (B) are representative of three independent experiments. Kinetic parameters were determined using equation 9; cBid dose-response data (B) were fit to hyperbolic curves (solid lines) using GraphPad Prizm software. (C) cBid-dependent dextran-Cascade Blue release induced by 70 nM (blue lines) and 210 nM (red lines) Bax at indicated concentrations of cBid. Bax was added at time zero; cBid was added 2 min prior to Bax. The data were used for quantification of kinetic parameters shown in (B). Dextran content is defined as the fluorescence signal at a given time point relative to the maximal fluorescence and quantified as described in Materials and Methods. See also Figure S5 demonstrating theoretical Bax dose-response curves. (D) Molecular scheme, modified from Figure 4B to include the data in (C) confirming that cBid activates Bax nonstoichiometrically, that is catalytically. Also, as k_1_ shows the same dependencies on [cBid] and [Bax] as k_2_, we infer that activated Bax enters into both tier I and tier II reactions. As k_1_ and k2 are linearly related to [Bax], the entire process shows no cooperativity with regard to Bax, and we conclude that Bax promotes the Catalyst Assembly reaction but cannot be the monomer M. Activated Bax could enter into the tier II reaction II, either as a catalyst or a direct participant. Data are representative of three independent experiments.

Based on the extensive literature correlating Bax oligomerization with MOMP, the monomer M in our reaction scheme (Figure 4) could have corresponded to Bax. To test this, we analyzed the dependence of the rate constants k_1_ and k_2_ on Bax concentration in our OMV system (Figure 5A). Surprisingly, both kinetic constants displayed a nearly linear Bax-dose dependence over a wide range of Bax concentrations (including the physiological range of 200–600 nM found in tumor cells; [9]), thus displaying an utter absence of both cooperativity and saturation. Thus, we were forced to conclude that in native membranes, Bax cannot correspond to the molecule M. Rather, Bax must act catalytically for the first reaction, promoting oligomerization of another molecule (M). Because we found that the kinetic constant for the second reaction, pore formation, was directly proportional to Bax concentration, we conclude that Bax is rate-limiting and non-cooperative in this reaction also. To illustrate this point, we generated theoretical curves predicting dose-dependencies in the case of Bax cooperativity (Figure S5). These curves modeling Bax behavior as an oligomer are parabolic and under no conditions could be quasilinear.

The lack of Bax cooperativity argues that Bax oligomerization is not a rate-limiting step in pore formation, as Lovell et al. also reported for liposomes [26]. From the standpoint of kinetics, Bax could either be a simple reactant or a catalyst in the pore formation reaction. Finally, the lack of saturability for both k_1_ and k_2_ implies that Bax does not need to interact with a stoichiometric “receptor” in the MOM, or at least that any such interactions are transient and not rate-limiting, even at high [Bax].

In contrast to these Bax dose-responses, with cBid the kinetic constants k_1_ and k_2_ showed plateaus, indicating saturability (Figure 5B). This argues that cBid has a limited number of binding sites in the MOM, consistent with prior studies showing that one or more proteins are required for the Bax-activation function of cBid in MOMs [28],[29]. It may seem paradoxical that k_1_ and k_2_ both showed saturable dependencies on [cBid] and linear dependencies on [Bax], but this is expected if activated Bax enters into both the catalyst assembly and pore formation reactions (tiers I and II), as portrayed in Figure 5D.

Recently it was reported that Bax exists in a dynamic equilibrium between free and membrane-associated forms, and Bcl-xL can shift this equilibrium towards the free cytosolic form (i.e., “retrotranlocate” Bax) [46]. Our previous data are consistent with this idea, in that only a fraction of the input Bax molecules become associated with OMVs after activation by cBid, and the even smaller amount of Bax association with OMVs that occurs spontaneously in the absence of cBid-induced activation can be blocked by Bcl-xL [47]. However, other groups reported that Bcl-xL and Bax can neutralize each other while remaining in the MOM [37],[48],[49]. Our data here do not discriminate between these possibilities and do not reveal whether Bax molecules, after initiating the assembly of the non-Bax catalyst, remain in the membrane or dissociate from it. Moreover, the activated Bax molecules that enter into the tier II reaction (pore formation; Figure 5D) are not necessarily the same ones that initiate the tier I reaction (catalyst assembly).

We next used kinetic analysis to analyze the interaction between cBid and Bax. By titrating cBid at two different Bax concentrations, we determined that at higher Bax concentrations, less cBid is required for MOMP (Figure 5B,C). This reciprocal relationship argues that pore formation requires a transient molecular collision between cBid and Bax, rather than a stoichiometric high-affinity reaction. (For two reactants in solution, simple mass action requires the probability of collision to be proportional to the product of the concentrations of the reactants. Here the reactions occur at or in a membrane, but a collision reaction would still entail an inverse relationship of reactant concentrations.)

Indeed, stoichiometric binding interactions between Bax/Bak and BH3-only proteins have been difficult (although not impossible) to detect (e.g., [26],[50]). This has prompted some to argue that Bax and Bak become activated in cells only because BH3-only proteins sequester and inactivate their anti-apoptotic partners (e.g., Bcl-2, Mcl-1, and Bcl-xL) (e.g., [51]). In contrast, our experiment provides a direct biochemical confirmation of the alternate hypothesis that cBid directly activates Bax by a “hit-and-run” (catalytic) mechanism [10],[52],[53]. A recent biophysical study, using EPR spectroscopy with synthetic membranes, reached a similar conclusion [54]. Together with the identification of an alternative BH3-domain binding site in Bax that could mediate this kind of Bax activation [50] as well as recent genetic experiments [55], our data help validate the “direct activation” model for MOMP.

### A Thermodynamic Transition Specifically Affects the Catalyst Assembly Reaction

**Figure 6:**
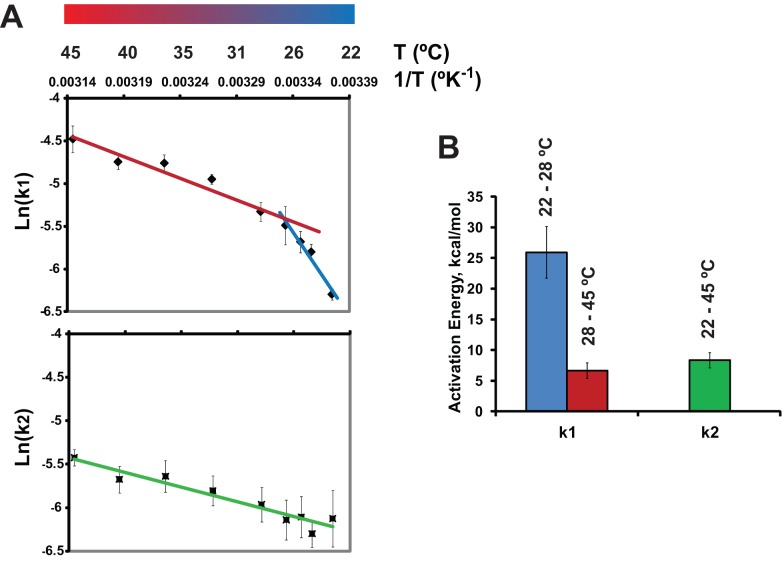
The first kinetic constant (k_1_), but not the second (k_2_), displays a transition (discontinuous slope) in the Arrhenius plot. A representative Arrhenius plot (A) and activation energies (E_a_) associated with the kinetic constants k_1_ and k_2_ (B). E_a_ values were determined from the slopes of replicate Arrhenius plots (similar to one shown in panel A). The two E_a_ values associated with k_1_ correspond to the temperatures above and below the transition point. The transition temperature is represented by the break-point on the Arrhenius plot (A) and was found to be 27.9±0.7°C, mean ± SE, *n* = 5. Data in (A) are means ± S.E of 4–9 replicates; data in (B) are means ± S.E of seven separate OMV preparations.

To confirm experimentally that reactions I and II in our model (Figure 4B) reflect distinct processes, we analyzed the temperature dependence of cBid/Bax-induced dextran release. We obtained values of k_1_ and k_2_ at each temperature by curve-fitting the kinetic data and plotted k_1_ and k_2_ in a standard Arrhenius representation (Figure 6). In this style of plot, the slope of the curve is proportional to the activation energy of the reaction. The plot for k_2_ was essentially linear; in other words, the activation energy for k_2_ (corresponding to the second reaction, pore formation) was temperature-invariant.

Strikingly, however, k_1_ exhibited two distinct slopes with a breakpoint at ∼28°C. Such a discontinuity in the Arrhenius plot is consistent with a transition in the biophysical state of the system (possibly in membrane microdomains) that facilitates formation of the catalyst (complex M*_n_) at temperatures above the breakpoint (see Discussion below). Importantly, these data support our kinetic model by confirming that k_1_ and k_2_ correspond to independent reactions with distinct energy barriers.

### Endogenous Outer Membrane Proteins Are Required for Bax-Dependent MOMP

Several proteins present in the MOM (other than Bcl-2 relatives) have been proposed to facilitate MOMP either by promoting Bax action [30],[31],[56]–[58] or by anchoring cBid [28],[29]. To determine whether proteins could account for the increased sensitivity of the native MOM to Bax, compared with liposomes, we inactivated MOM proteins using protease treatment, heat-inactivation, and chemical inhibition.

We first tested if pretreatment of OMVs with proteinase K affected cBid/Bax-induced dextran release, as it was reported that a similar treatment of isolated mitochondria inhibits Bax oligomerization [59]. We found that VDAC, an integral membrane protein, was resistant to digestion, as were Bif-1 (Figure S6B) and some other MOM proteins visible in Coomasie Blue-stained gels (unpublished data). In contrast, the MOM protein Tom20 was digested by proteinase K (Figure S6B). Surprisingly, proteinase K treatment had no effect on OMV permeabilization (Figure S6A). Thus, proteinase K-digestible proteins were dispensable for MOMP in our system.

**Figure 7:**
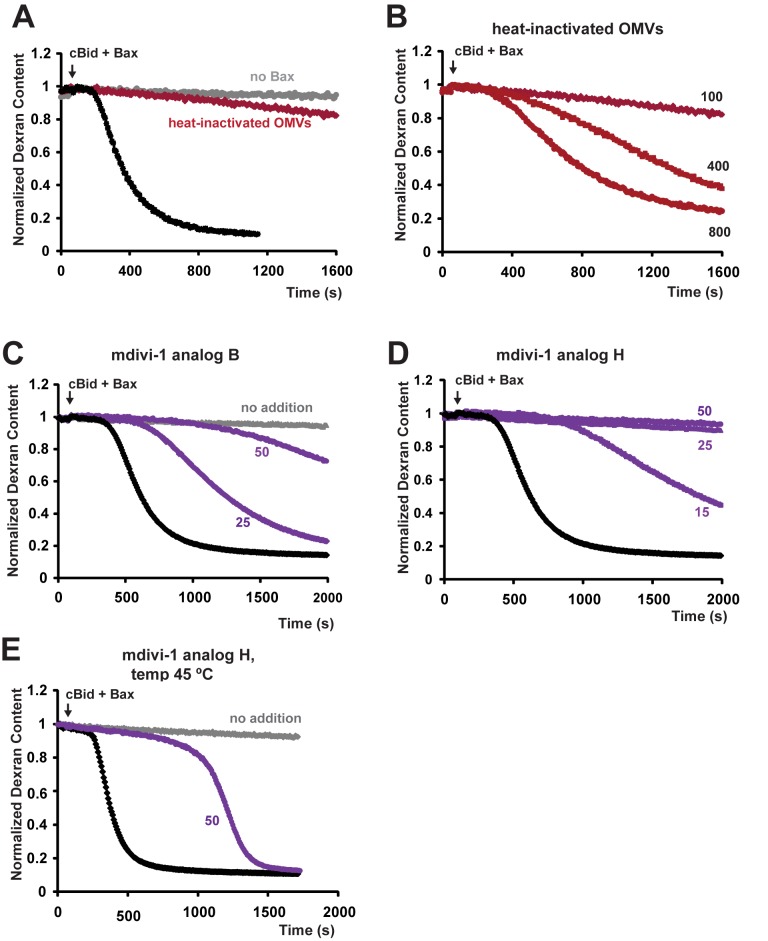
Bax-induced pore formation requires heat-labile protein(s) and is inhibited by mdivi-1 analogs. For heat-induced protein inactivation, OMVs were preincubated at 68°C for 10 min and equilibrated at room temperature. Dextran-fluorescein release induced by 100 nM Bax in the presence of 40 nM cBid was inhibited in heat-treated OMVs (A), but restored at higher (400–800 nM) Bax concentrations (B). OMVs were incubated with mdivi-1 analogs B and H for 5 min prior to the addition of Bax (C–E). Note that at 25°C, compound H (25–50 µM) completely inhibits dextran release (D); at 45°C, however, this compound produces merely a long lag phase without much effect on the rate of pore formation (E). Black lines, control OMVs; red lines, heat-treated OMVs; purple lines, OMVs treated with the mdivi-1 analogs. Arrows indicate additions of Bax; cBid was added 2–3 min prior to Bax. Data shown are representative of at least three independent experiments (see also Figure S6).

Next, we used heat treatment to inactivate MOM proteins more generally. According to a calorimetric study [60], most proteins in rat liver mitochondria undergo denaturation at temperatures just below 70°C. Accordingly, we incubated OMVs at 68°C for 10 min and then assayed for Bax-induced dextran release. This heat pretreatment completely eliminated the response to mixtures of cBid and Bax that ordinarily caused robust MOMP (Figure 7A), showing that MOM proteins are essential. (Heat treatment did not cause noticeable aggregation of OMVs, as determined by dynamic light scattering; unpublished data.) Such an effect could have been explained merely by the requirement of cBid for a heat-labile protein “receptor.” To examine this possibility, we repeated these experiments using another BH3-only protein, BimS, which induced Bax-dependent MOMP with very similar kinetics (Figure S6C). Additionally, we tested the effect of moderately elevated temperature (45°C), which can promote Bax activation in the absence of BH3-only proteins [61]. When Bax was pre-incubated alone at 45°C, it did not induce MOMP when added subsequently to OMVs. However, incubating OMVs at 45°C in the presence of Bax alone induced dextran release with biphasic kinetics like those seen at 25°C with cBid+Bax or BimS+Bax (Figure S6D). We found that 68°C heat-inactivation of OMVs also inhibited dextran release induced by BimS+Bax or by Bax at 45°C, albeit less completely than with cBid+Bax (Figure S6C). However, the inhibitory effect of preincubation at 68°C could be overcome by adding Bax at much higher concentrations (Figure 7B), suggesting that heat-labile proteins enhance the pore-forming activity of smaller input amounts of Bax. Together, these data demonstrate that functional MOM proteins are indispensable for cBid activity and also strongly potentiate Bax function.

### Dnm1-Inhibitory Compounds Block Bax-Dependent MOMP in OMVs by Targeting a Factor Involved in Catalyst Assembly

One candidate MOMP-enhancing protein we considered was Drp1. The potential role of this protein in MOMP has been studied both in cell biological [22],[62]–[64] and biochemical [30],[58] studies, but whether it plays an obligate role in MOMP is controversial. Downregulation of Drp1 in cells has been reported to delay cytochrome c release after the administration of apoptotic stimuli [22],[30],[65]. Recombinant Drp1 protein was shown to enhance the oligomerization of Bax in liposomes by inducing massive membrane remodeling [30]. Although Drp1 is not generally considered a resident MOM protein, it has been detected at various levels in some isolated mitochondria [22],[66]–[68].

Some compounds that inhibit Dnm1, the yeast ortholog of Drp1, also block Bax/Bak-dependent cytochrome c release from isolated mouse mitochondria [31], which suggests that Drp1 may be required for MOMP. We found that, in our rat liver OMV system, the Dnm1 inhibitors designated as mdivi-1 analogs “B” and “H” [31] inhibited cBid/Bax-induced OMV permeabilization (Figure 7C,D). Analog H also efficiently inhibited cBid/Bax-induced dextran release from Xenopus egg OMVs (unpublished data). An inactive analog (8L310s) had no effect in either system (unpublished data).

These results raised the possibility that Drp1 is the catalyst, M. Surprisingly, however, Drp1 was undetectable by immunoblot in rat liver OMVs, purified rat liver mitochondria (Figure S6E), and Xenopus egg mitochondria (Figure S6F), although strong Drp1 bands were seen in cytosol. Furthermore, the inhibitory effect of mdivi-1 analogs was observed also in the absence of added GTP and Mg^2+^ and was unaffected by the Mg^2+^ chelators EDTA and EGTA (unpublished data). Thus, GTP hydrolysis is not required for the functional target of mdivi-1 analogs. The in vitro membrane-remodeling activity of Drp1 described by Martinou and colleagues [30] requires ATP, not GTP. Our system lacks ATP and is unaffected by ATP addition (unpublished data). Thus, in OMVs, the mdivi-1 analogs apparently do not target a canonical Drp1 activity. It remains formally possible, but unlikely, that trace amounts of Drp1 in the isolated outer membrane act in an unconventional manner to promote MOMP.

### mdivi-1 Analogs and Membrane Fluidity Specifically Affect Catalyst Formation, Supporting the Two-Reaction Model

The mdivi-1 analog H did not block the binding of Bax to rat liver OMVs (Figure S3B,C), implying that this compound acts downstream of Bax recruitment to the membrane. The active mdivi-1 analogs were among the few agents we tested that could prolong the lag phase, suggesting that these compounds specifically inhibit reaction I. In contrast, elevated temperatures strongly facilitated reaction I. Figure 6 shows that a shift from 22°C to 45°C caused a 7-fold increase in the rate of catalyst formation (in the absence of mdivi-1 analogs), but only a 2-fold increase in the rate of pore formation. Our kinetic model therefore predicts that elevated temperatures would oppose the effects of mdivi-1 analogs, particularly with regard to the lag phase kinetics (catalyst assembly). To test this, we incubated OMVs at either 45°C or 25°C, in the presence or absence of mDivi-1 analog H. (Control experiments assured that the inhibitory compound was not trivially degraded during the 45°C incubation; unpublished data.) At 25°C, inhibition by analog H was virtually complete: no dextran release was observed during the 60-min experiment (Figure 7D and unpublished data). However, at 45°C, despite the presence of the inhibitor, pore formation began to occur after an extended lag (Figure 7E). Strikingly, the rate of dextran release (pore formation) was almost the same as in the absence of inhibitor.

This result could be explained by two different mechanisms, which are not mutually exclusive. On the one hand, the data are consistent with our kinetic model's prediction that elevated temperatures would facilitate assembly of the catalyst in reaction I, albeit slowly because of inhibition by the mdivi-1 compound. The other possibility is that incubation at 45°C could increase the lateral mobility of integrated Bax, allowing Bax to migrate slowly to putative foci of pore formation, despite inactivity of the catalyst. For either mechanism, we surmise that raising the temperature helps alleviate the effects of membrane crowding [69]. Regardless of the mechanism, these data support our two-reaction model by confirming that catalyst activation and pore formation are distinct, experimentally separable processes.

## Discussion

### Nature of the Pores

Our data show that very large dextrans and smaller dextrans were released with similar kinetics (Figure S4). A slightly shorter lag phase for 10 kDa dextrans may indicate that smaller pores are assembled faster than larger ones. This is perhaps consistent with electrophysiological studies describing dynamic low and high conductance states of Bax-dependent channels [70],[71]. However, Bax-induced pores permitting the unrestricted passage of very large dextran molecules (Figure S4A) are too large to be typical protein channels. Instead, our data, along with previous reports from others and ourselves, favor a mechanism for MOMP based on the formation of supramolecular lipidic pores [9],[27],. As opposed to the concept of discrete Bax channels [39],[70], lipidic pores would be at least partly framed by a toroidal lipid monolayer [72]. Such pores in the MOM could resemble the growing pores imaged in cBid/Bax-treated liposomes by Schafer et al. [28] using cryo-electron microscopy.

### Catalyst Assembly Is an Outer Membrane-Intrinsic Process that Does Not Involve Bax Oligomerization

Our cell-free OMV system, which is quite pure apart from the presence of attached ER (Figure 1) and lacks energy sources, is not expected to recapitulate cellular processes involving the translocation of molecules from other cellular compartments to the MOM. Nevertheless, our studies help establish MOM-intrinsic mechanisms of pore formation triggered by activated Bax. In particular, our experiments revealed an important mechanistic feature: the Bax-induced assembly of a catalyst that in turn enhances Bax pore formation. This event is intriguing, as it likely represents a new point of apoptotic regulation that has not been reproduced in liposomes.

Assembly of the oligomeric catalyst complex is relatively slow and highly cooperative. In contrast, Bax oligomerization is a “kinetically silent” (not rate-limiting) event, as shown by a linear dependence of the kinetic constants k_1_ and k_2_ on Bax concentration (Figures 4 and S5). Many studies, including our own, have observed that Bax/Bak oligomerization is correlated with MOMP, but it has never explicitly been shown that oligomerization is a requisite event. Our data showing a lack of Bax cooperativity now argue that, on the contrary, Bax oligomerization does not contribute to MOMP kinetics. Instead, we propose that integrated Bax monomers are the active agent, assisted by the catalyst complex.

### How Might the Catalyst Facilitate Pore Formation?

The catalyst could function via either of two general mechanisms, which are not mutually exclusive: (1) facilitating the pore formation mechanism per se and thereby perhaps lowering the threshold concentration of Bax required for pore formation, or (2) facilitating the accumulation of integrated Bax in putative membrane microdomains, which might serve as preferential sites of pore formation.

Evaluating these possibilities will require further study. In the meantime, there are reasons to suggest that the catalyst acts at least via mechanism 2. Firstly, activated Bax can permeabilize liposomes and thus has an intrinsic pore-forming function. Therefore, a partitioning of Bax into local domains is the simplest hypothesis that could explain an enhanced rate of pore formation. Secondly, this notion is also supported by the observation that high concentrations of Bax can overcome the block to MOMP caused by heat-inactivation of MOM proteins (Figure 7B). However, the catalyst could also act by mechanism 1. Indeed, we found that intermediate concentrations of mdivi-1 analog H affect both the lag and rapid kinetic phases (Figure 7C,D), arguing that the catalyst also directly enhances the pore formation process.

An intriguing possibility is that the catalyst could remodel the membrane, inducing a localized change in curvature. Perhaps, in a manner analogous to the activity recently reported for Drp1 in liposomes [30], the mdivi-1-sensitive catalyst could induce a local membrane deformation that facilitates the concentration of Bax into membrane microdomains with altered curvature. Furthermore, such a remodeling event could add stress to the membrane, reducing the energy barrier for pore formation induced by Bax. In this way, the catalyst could reduce the local threshold Bax concentration for pore formation (i.e., act via mechanism 1).

Supporting the idea of a membrane-remodeling event is the observation of a sharp temperature-dependent change in activation energy for k_1_ that is reminiscent of a membrane phase transition (Figure 6). This led us to hypothesize that at temperatures above the transition point at ∼28°C, altered lipid packing facilitates activation of the catalyst molecule. Surprisingly, however, MOMs did not exhibit a large-scale membrane phase transition (Figure S7). Taking these observations together, we surmise that MOMs undergo Bax-dependent lipid rearrangements that are limited to microdomains and thus would not be measurable in the bulk membrane. Elevated temperatures would facilitate changes in the lipid packing in these small domains and thereby help promote activation of the catalyst molecule.

Bax could be involved in a concerted remodeling of such membrane microdomains, both in its role as inducer of catalyst activation (tier I in Figure 5D) and in its role in the pore formation reaction (tier II). Previous studies revealed the intrinsic ability of activated Bax to destabilize membranes. For example, activated Bax was observed by cryo-EM to increase the curvature of membrane regions in artificial lipid vesicles [28]. Remodeling mechanisms based on protein-induced curvature have been observed in other biological membranes [77],[78].

### What Is the Identity of the Oligomeric Catalyst Protein?

Despite the inhibition of MOMP by mdivi-1 analogs, our data did not support a requirement for Drp1. We found that pore formation in OMVs was independent of ATP and GTP. Thus, if Drp1 were involved, its action would involve a previously unreported type of activity. We attempted to observe an activity of exogenous recombinant Drp1, but this protein did not stimulate MOMP in OMVs, nor did it restore MOMP to heat-inactivated OMVs (unpublished data). More significantly, endogenous Drp1 protein was below the limits of detection in rat liver OMVs and mitochondria and in Xenopus OMVs (Figure S6E,F), which all displayed similar biphasic MOMP kinetics. Our results suggest that, with regard to MOMP, the true vertebrate target of mdivi-1 analogs is not Drp1. However, we cannot formally exclude the (unlikely) possibility that the catalyst manifests a noncanonical activity of a form of Drp1 that failed to be detected by the antibody.

We considered one other candidate for the catalyst: the Bax-interacting protein Bif-1/Endophilin B1. Like Drp1, Bif-1 can form high-order oligomers and remodel membranes in vitro [58],[79]. As with Drp1, we hoped to see an effect of adding exogenous protein. However, recombinant Bif-1 did not stimulate MOMP when added to rat liver OMVs and even had a slightly inhibitory effect on MOMP when included during reconstitution of proteoliposomes from Xenopus OMV lipids and proteins ([28]; unpublished data). More significantly, although a small fraction of Bif-1 was found in rat liver OMVs in a protease-resistant form (Figure S6B), Bif-1 was not detectable in Xenopus OMVs (Figure S6G; the antibody did recognize Bif-1 strongly in Xenopus cytosol) As Xenopus OMVs respond to Bax with kinetics very similar to those of rat liver OMVs, the absence of detectable Bif-1 suggests that this protein is not the catalyst molecule M. (However, the same caveat applies as stated above for Drp1.) The molecular identification of the catalyst awaits further investigation.

In conclusion, our studies revealed some surprising aspects of Bax-induced pore formation in native mitochondrial outer membranes. In particular, our data were inconsistent with an involvement of oligomeric Bax and were also inconsistent with a requirement for Drp1. Moreover, our kinetic studies revealed a two-tiered system of reactions that is not reproduced by simplified liposome models. In native MOMs, Bax induces the relatively slow formation of a multimeric catalyst complex that strongly facilitates the formation of supramolecular membrane pores. Assembly of this catalyst complex was influenced by temperature in a way that suggested dependence on membrane biophysical properties. We propose that the catalyst complex acts both to accelerate the redistribution of membrane-integrated Bax and to facilitate the process of pore formation directly, perhaps through a membrane-remodeling event. The catalyst, although still unidentified, could represent a novel point of regulation for apoptotic cell death and could represent an additional therapeutic target for diseases involving aberrant cell death or survival.

## Materials and Methods

### Isolation of Mitochondria and Outer Membrane Vesicles (OMVs)

Mitochondria were isolated from the livers of male Sprague-Dawley rats by standard differential centrifugation techniques [80]. The isolation buffer contained 210 mM mannitol, 70 mM sucrose, 10 mM HEPES-KOH (pH 7.4), 2 mM EGTA, and 0.1% bovine serum albumin (essentially fatty acid free, Sigma). Isolated mitochondria were further purified in a step gradient of iodixanol (OptiPrep, AxisShield-Sigma) as described previously [81] with some modifications. The mitochondrial pellet was resuspended in 36% iodixanol diluted in SHE buffer (250 mM sucrose, 10 mM HEPES-KOH, pH7.4, 2 mM EGTA), to a final volume of 9–10 ml. The iodixanol gradients, consisting of 3 ml SHE, 5 ml 17.5% iodixanol, 5 ml 25% iodixanol, and ∼1.5 ml of the mitochondrial suspension in 36% iodixanol, were prepared in six 16 ml tubes. The gradients were centrifuged at 50,000×*g* for 2 h. Purified mitochondria were recovered from the 17.5%/25% interface. A fraction formed on the SHE/17.5% interface contained light mitochondria contaminated with endoplasmic reticulum (ER). This fraction was collected for comparison with the purified mitochondrial fraction and OMVs. In addition, a crude ER fraction (microsomes) was isolated from the rat liver homogenate as described [81] and used for ER marker assays. For preparation of OMVs, purified mitochondria were diluted 7–8 times in a hypotonic buffer (10 mM KOH, pH 7.4, 0.5 mM EGTA, 4 mM KCl) and incubated for 10 min. After hypotonic treatment, mitochondria were centrifuged at 12,000×*g* for 10 min and the pellet was resuspended in 2 ml of the hypotonic buffer supplemented with 5 mg of either 70 kDa dextran-fluorescein (Sigma) or 10 kDa dextran-cascade blue (Invitrogen). The suspension was homogenized in a 7 ml glass Dounce homogenizer using a tight-fitting pestle (40–50 strokes). The homogenate volume was then adjusted to 6 ml and OMVs were purified in iodixanol step gradients prepared in three 16 ml tubes. Each tube contained 2 ml of the homogenate, 5.5 ml 8% iodixanol, 5.5 ml 17.5% iodixanol, and 1.5 ml 25% iodixanol. The gradients were centrifuged at 50,000 *g* for 2 h and OMVs were collected from the 8%/17.5% interface. Unincorporated fluorescent dextran was separated from OMVs by the 8% iodixanol layer. Remaining mitochondria partially devoid of the outer membrane (mitoplasts) banded on the 17.5%/25% interface. This fraction was used in analyses of the purity of OMVs. Additional removal of external dextran was achieved by diluting OMVs 10-fold in the hypotonic buffer and concentrating them by centrifugation at 100,000×*g* for 20 min. The pellet was resuspended in 200 µl of the hypotonic buffer. Typical protein concentration in OMV suspension was ∼2 mg/ml as determined by BCA assay (Pierce). All isolation steps were performed at 4°C. *Xenopus* Egg OMVs loaded with 70 kDa dextran-fluorescein were prepared as described previously [9].

### Generation of Recombinant Proteins and BH3 Peptides

Human Bcl-xL and cleaved human Bid (cBid) were generated as described [9]. For some experiments, we used a full-length Bid construct containing a thrombin digestion site in place of the caspase cleavage site [82], or alternatively, commercial caspase-8-cleaved Bid (R&D Systems); these were equally active. Full-length human Bax was produced essentially as described by Suzuki et al. [83]. Recombinant BimS protein was a gift from Dr. Frédéric Luciano (then of the Sanford-Burnham Medical Research Institute). This protein was prepared essentially as described for BimEL [84], with induction by 1 mM IPTG for 1 h at 37°C (Frédéric Luciano, personal communication). Bad and Noxa BH3 peptides [21] were obtained from AnaSpec.

### Chemical Inhibitors of Dnm1

Analogs of mdivi-1 inhibiting the GTPase activity of Dnm1, the yeast ortholog of Drp1, were obtained from BioNet, UK. The active compounds used were 7L-365S [3-(2,4-dichloro-5-isopropoxyphenyl)-2-sulfanyl-4(3H)-quinazolinone] and 8L-309S [7-chloro-3-(2,4-dichloro-5-isopropoxyphenyl)-2-sufanyl-4(3H)-quinazolinone], which correspond to mdivi-1 analogs B and H, respectively, in the original report [31]. An inactive analog 8L-310S [6-chloro-3-(2,4-dichloro-5-isopropoxyphenyl)-2-sulfanyl-4(3H)-quinazolinone] was used as a negative control.

### Preparation of Liposomes

To prepare defined liposomes, the following phospholipids were used: phosphatidylcholine (PC) and phosphatidylethanolamine (PE) from chicken egg, phosphatidylinositol (PI) and phosphatidylserine (PS) from soybean, and cardiolipin (CL) from bovine heart. The lipids (in chloroform solution) were obtained from Avanti Polar Lipids and mixed in the molar ratio found in isolated mitochondria [9]—that is,, PC∶PE∶PI∶PS∶CL = 47∶28∶9∶9∶7 (mol/mol). When CL was omitted, the lipid ratio was PC∶PE∶PI∶PS∶CL = 54∶28∶9∶9 (mol/mol). After lipid mixing, chloroform was removed by evaporation under an argon stream. The lipid film was lyophilized for 2–3 h. Dry lipids were resuspended in KHE buffer (150 mM KCl, 10 mM HEPES, pH 7.4, 0.5 mM EGTA) containing 70 kDa dextran-fluorescein (1 mg/ml) and subjected to five freeze/thaw cycles. Unilamellar liposomes were prepared by extrusion through 200 nm polycarbonate filters using a Mini-Extruder (Avanti Polar Lipids). Unentrapped dextran-fluorescein was removed by gel filtration using a Sephacryl S-500 HR column (GE Healthcare Bio-Sciences). In some experiments, defined liposomes of the indicated composition were prepared by a detergent removal method as described previously [28]. To prepare liposomes from extracted mitochondrial lipids, phospholipids were extracted from isolated rat liver mitochondria according to the published protocol [85]. The extracted lipids were processed as described above and liposomes were formed by the extrusion method.

### Fluorescence Assays to Measure Kinetics of Membrane Permeabilization and Bax Insertion

OMVs loaded with Cascade Blue- or fluorescein (FITC)-labeled dextran (10 and 70 kDa, respectively) were incubated in the KHE buffer in the presence of specific antibodies that bind and quench the fluorophores. The final concentrations of anti-Cascade Blue and anti-fluorescein antibodies (Invitrogen) were ∼300 and ∼80 µg/ml, respectively. The type of dextran used is specified in the figure legends. Both sets of fluorophore/antibody gave essentially the same results. Fluorescence measurements were performed with a POLARstar Omega microplate reader (BMG Labtech) using 400 nm excitation and 420 nm emission filters for cascade-blue and 485 nm excitation and 520 nm emission filters for fluorescein. The assay volume was 100 µl. Alternatively, cascade blue fluorescence was monitored in a FluoroMax-2 spectrofluorometer (Horiba Jobin-Yvon) using a 200 µl quartz cuvette; the wavelengths were set at 400 nm excitation/418 nm emission. Both the cuvette- and microplate-based assays produced the same results. Addition of the antibodies to intact OMVs typically resulted in a small (2%–10%) decrease in the fluorescence signal reflecting quenching of residual external dye. Membrane permeabilization was then induced by cBid and Bax (unless indicated otherwise) and ensuing dextran release was measured continuously for 20–35 min at 25°C (or at specified temperatures). OMV protein concentration in the assay buffer was 0.2–0.3 mg/ml for rat liver and 0.05 mg/ml for Xenopus egg OMVs. Triton X-100 (0.05%–0.1%) was added to set a baseline for complete permeabilization of the vesicles. Normalized dextran content in OMVs was quantified using the equation: F = (F_t_−F_triton_)/(F_0_−F_triton_), where F_t_ is the fluorescence measured at a given time, F_triton_ is the fluorescence measured in the presence of Triton X-100, and F_0_ is the initial (maximal) fluorescence of the vesicles. OMVs were stored on ice in the dark and used within 1–2 d after preparation. No spontaneous leakage of the dyes from the vesicles was detected for at least 3 d after preparation. The same membrane permeabilization assay was performed with dextran-fluorescein loaded liposomes.

Bax membrane insertion was measured using Bax labeled with NBD dye (IANBD amide, Invitrogen) as described previously [26] with some modifications. Bax contains two endogenous cysteine residues that react with NBD. For labeling, freshly prepared NBD solution was added to ∼400 µl Bax (∼6 µM) in a labeling buffer. Labeling buffer was prepared as described [26], except that the CHAPS concentration was reduced to 0.25%. The final concentration of NBD in the labeling reaction was ∼60 µM. The reaction mixture was incubated under constant stirring in the dark for 1.5 h at room temperature. Unbound dye was removed by gel-filtration using Sephadex G-25 columns (PD-10, Pharmacia Biotech). The protein was eluted using the labeling buffer without CHAPS. Fractions with labeled Bax were identified by measuring tryptophan fluorescence (excitation 280 nm/emission 340 nm) and NBD (excitation 475 nm/emission 530 nm) in an LS50B spectrofluorometer. The membrane-permeabilizing activity of Bax-NBD was the same as unlabeled Bax but declined significantly after freeze/thaw or storage for over 24 h at 4°C. For membrane insertion measurements, freshly prepared Bax-NBD was added to OMVs, and NBD fluorescence was measured in the POLARstar-Omega plate reader using 485 nm excitation and 520 nm emission filters. The assay conditions were the same as described for the dextran release measurements.

### Bax Binding to OMVs

OMVs were incubated with cBid and Bax as described above for the dextran release measurements. The assay mixture was then placed on ice and subjected to float-up density gradient centrifugation as described [21] or with an improved technique: the sample (200 µl) was mixed with the equal volume of 50% iodixanol in SHE buffer. This mixture was overlaid with 1 and 0.4 ml layers of 17.5% iodixanol and SHE. The gradients were centrifuged in a microcentrifuge at 16,000×g for 70 min (at 4°C). The membranes were recovered from the SHE/17.5% iodixanol interface, placed into a 0.1 µm microfiltration unit (Millipore), and collected from the retentate after filtration (centrifugation at 12,000×g for 8–10 min). Bax content in the membranes was determined by immunoblotting. Tom20 was analyzed as a loading control. Densitometry analysis of Western blots was performed using ImageJ software (NIH). Bax band intensities were corrected for loading differences. Changes in Bax content were normalized to control (loaded on the same gel). Time-dependent Bax binding to OMVs was normalized to the Bax signal obtained at the first time point (∼0.1 min after Bax addition in the presence of cBid).

### Membrane Fluidity Measurements

Isolated mitochondria and OMVs (0.2 mg/ml) were incubated with 10 µM DPH (Sigma), a hydrophobic probe for fluorescence polarization (FP) measurements [86]. DPH-labeled samples were equilibrated at each given temperature for 10 min and FP was measured in the POLARstar-Omega plate reader using 355 nm excitation and 430 nm emission filters.

### Kinetic Analysis of Dextran Release Curves

Based on experimental evidence (discussed in Results), we concluded that the release of fluorophore-conjugated dextran per se from vesicles as well as its quenching by appropriate antibodies are rapid and non-rate-limiting processes that could thus be omitted from the model. To facilitate the kinetic modeling, we made some reasonable simplifying assumptions. First, we assumed that the rate of dextran release is proportional to the rate at which vesicles become permeabilized. In turn, this is proportional to the rate of pore formation (see text). These assumptions imply that the vesicles exist only in two states: empty and full. Thus, for the purposes of modeling, the vesicles are analogous to biochemical entities, which are also binary: either reacted or unreacted. Importantly, this enabled us to use methods established for modeling biochemical kinetics. The simple reaction schemes that we examined at first (Figure 4, panels 1,2) clearly did not fit the observed kinetics of dextran release, which displayed a pronounced initial lag. However, we found that a somewhat more complex model involving two coupled reactions (Figure 4A, panel 3) produced an essentially perfect fit to the experimental data. In this model, Reaction I entails the assembly of a multimeric complex. This complex serves as a catalyst for Reaction II, vesicle permeabilization (which according to our assumptions is physically equivalent to the formation of a dextran-permeable membrane pore). Fortunately, we could derive an exact mathematical solution for the theoretical curves. Reaction II is a pseudo-first-order process whose rate is proportional to the concentration of the intact vesicles:
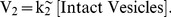
(1)The pseudo-constant of this reaction k_2_^∼^ is proportional to the concentration of the catalytic complex C formed in Reaction I:
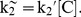
(2)Next, we consider Reaction I, which consists of two steps, in the first of which a monomeric molecule M is activated to form M*. This relatively slow event is followed by a rapidly reversible second step, the multimerization of activated monomer M* to form the catalyst complex M*_n_. The activation step is a first-order reaction, and therefore the integral form of the rate law is the exponential function:
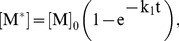
(3)where k_1_ is the kinetic constant and [M]_0_ is the initial concentration of the inactive monomer M. The second step, multimerization of M*, is governed by the thermodynamic mass action law:
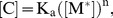
(4)where K_a_ is the thermodynamic equilibrium constant and n is the degree of multimerization.

Combining Equations 2–4, we obtain the expression for k_2_^∼^:
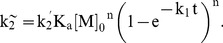
(5)Curve-fitting analysis of the experimental data for dextran release (see below) would produce estimates for the rate constant k_1_ and the coefficient, k_2_′ K_a_ [M]_0_^n^, but not for the individual components (k_2_′, K_a_, or [M]_0_). Thus, we must replace this coefficient with a combination constant, k_2_, obtaining the following equation:
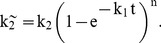
(6)The equation describing dextran release is the standard solution for this kind of rate law:
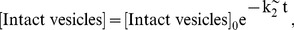
(7)where [Intact vesicles]_0_ is the initial concentration of vesicles. The fluorescence of the entrapped dextrans corresponds essentially to the fraction of intact vesicles, multiplied by the fluorescence intensity of the labeled dextrans. In practice, we also have to take into account the background fluorescence. The measured fluorescence, F(t), at time t can thus be rewritten:
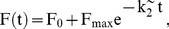
(8)where F_0_ is the background fluorescence at time zero and F_max_ is the change in fluorescence intensity after complete quenching (i.e., after addition of detergent). Finally, by substituting the formula for k_2_^∼^ from equation (6) and adding an additional term for a constant rate (k_3_) of background leakage that we observed even in the control curves, we obtain the following equation, which was used to fit the experimental data:
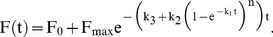
(9)In summary, this kinetic analysis identified k_1_ and k_2_ as parameters quantitatively describing the process of MOMP. They reflect, respectively, the rate of catalyst assembly and the rate of vesicle permeabilization. In a dextran release curve (e.g., Figure 4A), k_1_ determines the duration of the lag phase, while k_2_ is reflected in the maximal slope during the rapid phase.

### Respiration-Based Mitochondrial Membrane Permeability Assay

To determine the kinetics of MOMP in intact mitochondria, we measured Complex IV (cytochrome c oxidase)-dependent respiration in the presence of exogenous cytochrome c and a Complex III inhibitor, using a Clark-type electrode (Hansatech). Added cytochrome c was continuously reduced via the NADH/cytochrome b5 reductase system, with NADH as the source of reducing equivalents [42]. Under these conditions, the rate of respiration depends on cytochrome c influx through the MOM and can therefore be used as a measure of MOMP. Specifically, isolated rat liver mitochondria (0.4 mg/ml) were incubated in a KCl-based respiration buffer containing 125 mM KCl, 10 mM HEPES (pH 7.4), 0.5 mM KH_2_PO_4_, 50 µM EGTA, 0.3 µM FCCP, and 10 mM succinate. Succinate-supported uncoupled respiration was measured for 3–4 min followed by addition of 2 µM myxothiazol, the Complex III inhibitor. Complex IV-dependent respiration was then stimulated by NADH (2 mM), cytochrome c (80 µM), and cBid/Bax at indicated concentrations. Respiration was measured until O_2_ exhaustion or termination of the reaction by addition of the Complex IV inhibitor, KCN (0.5 mM).

### Electron Microscopy

5–10 µl of the OMV suspension was applied onto a carbon-coated cooper 300 mesh grid (Ted Pella, Inc.). The sample was negatively stained with 2.0% aqueous uranyl acetate for ∼30 s. Excess of staining solution was removed and the grids were air-dried. The vesicles were visualized in an electron microscope (H-600A; Hitachi) and images were acquired with a cooled 11.2-Megapixel CCD camera (SIA-L9C; Scientific Instruments and Applications, Duluth, GA) using Maxim DL software, v. 5.2 (Diffraction limited, Ottawa, Canada).

### Western Blot Analysis

Samples of mitochondria, OMVs, mitoplasts, and ER (1.2–10 µg) were run on NuPage 4%–12% Bis-Tris gels (Invitrogen) at 200 V for 45 min. Proteins were electrotransferred to nitrocellulose membrane (BioRad) at 30 V for 1 h. The membranes were stained with antibodies to cytochrome c oxidase subunit IV (COX IV, 1∶1,000 dilution; Invitrogen), VDAC (1∶2,000; Calbiochem), and calnexin (1∶100; Chemicon). Other antibodies used were to Tom20 (1∶1,000; Santa Cruz Biotechnology), Bif-1 (1∶200; Imgenex), Bax-N20 (1∶1,000; Santa-Cruz), and Drp1 (1∶500; BD-Biosciences). The secondary antibodies were horseradish peroxidase-conjugated anti-mouse- or anti-rabbit-Ig antibodies (1∶2,000 dilution; Amersham). Protein bands were detected using either ECL reagent (Amersham) or SuperSignal West Femto reagent (Thermo Scientific) and X-ray film.

### Enzyme Activity Measurements

Succinate dehydrogenase (SDH) activity was measured spectrophotometrically using coenzyme Q1 and 2,6-dichlorophenolindophenol (DCPIP) (modified from [87]). Samples of mitochondria, mitoplasts and OMVs were permeabilized with 0.1% Triton X-100. The reaction buffer contained 20 mM HEPES (pH 7.5), 10 mM succinate, 60 µM coenzyme Q1, 80 µM DCPIP, and 1 mM KCN. Kinetics of succinate oxidation coupled with DCPIP reduction was measured as DCPIP absorbance decrease at 600 nm in the POLARstar-Omega plate reader. No changes in DCPIP absorbance were detected in the presence of 50 µM 2-thenoyltrifluoroacetone, a specific SDH inhibitor. Final concentrations of the samples were 0.01–0.02 mg/ml; assay volume was 100 µl. SDH activities (the rates of DCPIP reduction) were normalized per mg of protein. The reagents were from “Sigma.” Monoaminooxidase (MAO) activity was measured fluorometrically using Amplex Red MAO assay kit (Invitrogen) and the manufacturer's protocol. The kinetics of the reaction were measured in the plate reader with 544 nm excitation and 590 nm emission filters. Rates obtained in the presence of specific MAO inhibitors (provided in the kit) were subtracted as background. MAO activities were normalized per mg of protein. To quantify the level of enrichment in corresponding markers, SDH and MAO activities in OMVs and mitoplasts were normalized to their activities in the whole mitochondria from which they were prepared.

## Supporting Information

Figure S1 Rat liver OMVs, whole mitochondria, and Xenopus Egg OMVs display biphasic kinetics of MOMP, while cBid/Bax-induced permeabilization of liposomes shows simple one-phase kinetics. (A) Rat liver OMVs loaded with dextran-fluorescein were permeabilized by 20 nM cBid and 100 nM Bax, but not by 2 µM cBid alone. (B) Xenopus Egg OMVs loaded with dextran-fluorescein were permeabilized either by 20 nM cBid (20 nM) and Bax at indicated concentrations (black lines) or by cBid alone (120 nM, grey line). (C). Endogenous Bax was not detected in rat liver mitochondria and OMVs. Rat cell (1RB_3_AN_27_) lysate (a positive control), OMVs, and mitochondria were loaded at 30 µg; recombinant Bax (rBax) was loaded at 0.5 and 2 ng (D) Black lines, liposomes formed from extracted mitochondrial lipids were loaded with dextran-fluorescein and permeabilized by cBid (40 nM) and Bax at indicated concentrations; grey line, cBid was added in the absence of Bax. Arrows indicate additions of cBid and Bax. (E) Respiration-based continuous measurements of MOMP in isolated rat liver mitochondria. Bcl-xL (2 µM) inhibits cBid/Bax-induced MOMP in whole mitochondria. Other details are as specified in Figure 2E.
(EPS)

Figure S2 De-repressors of anti-apoptotic proteins do not affect MOMP kinetics in OMVs. OMVs were pretreated for 3–5 min with peptides (50 µM) corresponding to the BH3 domains of de-repressor proteins Bad and Noxa (A) or the BH3 mimetic drug ABT-737 (B). Arrows indicate additions of Bax (50 nM) and delayed addition of ABT-737 (3.5 µM) in the presence of Bcl-xL (1 µM). cBid and Bcl-xL (when present) were added 2–3 min prior to Bax. ABT-737 reversed the inhibitory effect of Bcl-xL (blue line) but had no effect on dextran-fluorescein release in the absence of added anti-apoptotic proteins (black line). (C) In the presence and absence of Bcl-xL, permeabilization of liposomes occurs without a lag-phase. cBid/Bax-induced dextran release was partially inhibited by 100 nM Bcl-xL. Arrows indicate additions of cBid (40 nM), Bax (200 nM), and an increased amount of cBid (100 nM) that reverses the inhibitory effect of Bcl-xL.
(EPS)

Figure S3 Bax association with OMVs is an early upstream event. (A–C) Bax recruitment to the MOM was blocked by Bcl-xL but not by Dnm1 inhibitor. OMVs were incubated with BAX in the presence or absence of cBid (40 nM) for 20 min (panels A–C) or for the indicated times (panels D,E). Bax concentration was 100 nM (B–C), 75 nM (D–F), or as indicated (A). OMVs were then recovered by float-up density gradient centrifugation and Bax content was analyzed by immunoblotting. Tom20 was analyzed as a loading control. (A–C) Bax binding to OMV was cBid-dependent and inhibited by Bcl-xL; compound H had less pronounced inhibitory effects. When present, Bcl-xL (2 µM) and compound H (50 µM) were added 5 min before Bax addition. (C) Quantification of immunoblots by densitometry was performed as described in Materials and Methods. Data presented are means ± S.D. from three immunoblots. (D, E) Bax is recruited early in the MOMP process, before the end of the lag phase. Samples were collected at indicated time points corresponding to different phases on the dextran release curves: the lag phase, the onset of dextran release, and nearly complete permeabilization. A representative cBid/Bax-induced dextran-fluorescein release curve from this set of experiments is depicted in panel F. (E) Quantification of immunoblots by densitometry. Data are presented as means ± S.E. from 3–4 immunoblots generated in three independent experiments. Black line, OMVs were incubated in the presence of cBid and Bax; grey line, no cBid was added.
(EPS)

Figure S4 Small (10 kDa) and larger dextrans (70 and 500 kDa) are released from OMVs by cBid/Bax with similar kinetics, showing that the pores are very large and dextran diffusion is not rate-limiting. OMVs were loaded with dextran-fluorescein of indicated sizes. (A) Bax (50 nM) was added at time zero; cBid (40 nM) was added 2 min prior to Bax. Note that the release kinetics for 70 and 500 kDa dextrans were indistinguishable. (B) Quantification of the rate constants for 10 kDa and 70 kDa dextrans. Data are presented as means ± S.E. of four independent experiments.
(EPS)

Figure S5Illustrative dose-response curves simulating the catalytic action of monomeric versus hypothetical dimeric and tetrameric forms of Bax. The rate constant of a catalyzed reaction (e.g., k_1_) is proportional to the concentration of catalyst (e.g., Bax). For monomeric, dimeric, and tetrameric Bax, respectively, equations 1–3 are as follows (in accordance with the thermodynamic mass action law):
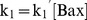
(1)

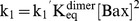
(2)

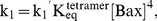
(3)where K_eq_ are association constants of BAX multimerization. The three panels correspond to various scenarios with K_eq_ values ranging from high to low (top to bottom).(EPS)

Figure S6 Treatment of OMVs with proteinase K had no effect on kinetics of cBid/Bax-induced dextran release, but 68°C heat treatment of OMVs inhibited Bax-induced dextran release; Drp1 was undetectable in highly enriched mitochondrial fractions. (A) OMVs (24 µg of protein) were incubated with 80 or 160 µg/ml proteinase K for 20 min at 37°C, followed by an addition of 0.5 mM PMSF (phenylmethanesulfonylfluoride). OMVs were washed twice by centrifugation and resuspended in the standard assay buffer. Dextran-fluorescein release from control (blue lines) and proteinase K-treated OMVs (green and red lines) was induced by 40 nM cBid and 100 nM Bax. (B) Western blot analysis of selected protein degradation by proteinase K. (C) Dextran release from control (black lines) and 68°C heat-inactivated OMVs (red lines) was induced by 100 nM Bax in the presence of 80 nM BimS; note that BimS produced a lag similar to that seen with cBid. At elevated temperatures (45°C), Bax (100 nM) caused dextran release in the absence of cBid or BimS, after a lag phase (D). Arrows indicate additions of Bax; when present, BimS was added 2 min prior to Bax. (E) Western blot analysis did not detect Drp1 in rat liver OMVs and isolated mitochondria. Samples of OMVs and rat liver mitochondria were loaded at 10 µg of protein. As positive controls, cell lysate from rat neural cells (1RB_3_AN_27_) was loaded at 10 µg of protein, and purified recombinant Drp1 (rDrp1) was loaded at 4 and 10 ng. (F, G) Drp1 and Bif-1 were readily detected in Xenopus Egg cytosol but not in the mitochondrial fractions. Mitochondrial and cytosolic fractions isolated from Xenopus eggs were loaded at 30 µg (F) or 10–14 µg (G) of protein. Recombinant Bif-1 (rBif-1) was loaded at 27.5 ng. No Bif-1 band was detected in Xenopus Egg OMVs (G).
(EPS)

Figure S7 Effect of temperature on membrane fluidity. (A) Model membrane system exhibiting a phase transition, reflected in a marked change in membrane fluidity. To assay membrane fluidity, DPH fluorescence polarization (FP) was measured in artificial liposomes prepared from 1-myristoyl-2-palmitoyl-sn-glycero-3-phosphocholine (MPPC), a lipid with a well-defined transition temperature (35°C; Avanti Polar Lipids, Inc.). A sharp change in FP was observed in the 35–37°C range, reflecting a large-scale gel-to-liquid crystalline phase transition. Data shown are replicate samples (black and grey lines). (B) FP was measured in DPH-labeled isolated mitochondria (blue line) and OMVs (red line) as a function of temperature. FP is reciprocally related to membrane fluidity. Note that the membranes displayed a gradual increase in membrane fluidity as the temperature was increased. OMVs displayed higher FP values than total mitochondrial membranes (likely reflecting higher cholesterol content). Data shown are average of three replicate samples.
(EPS)

## Abbreviations

cBid: cleaved Bid
DCPIP: 2,6-dichlorophenolindophenol
DPH: 1,6-diphenyl-1,3,5-hexatriene
ER: endoplasmic reticulum
FCCP: 4-(trifluoromethoxy)-phenylhydrazone carbonyl cyanide
FITC: fluorescein isothiocyanate
FP: fluorescence polarization
GAPDH: glyceraldehyde phosphate dehydrogenase
MAO: monoaminooxidase
MOM: mitochondrial outer membrane
MOMP: mitochondrial outer membrane permeabilization
MPPC: 1-myristoyl-2-palmitoyl-sn-glycero-3-phosphocholine
NBD: N,N′-dimethyl-N-(iodoacetyl)-N′-(7-nitrobenz-2-oxa-1,3-diazol-4yl)ethylenediamine
NIH: National Institutes of Health
OMVs: outer membrane vesicles
SDH: succinate dehydrogenase

## References

[1] Newmeyer DD, Farschon DM, Reed JC (1994) Cell-free apoptosis in Xenopus egg extracts: inhibition by Bcl-2 and requirement for an organelle fraction enriched in mitochondria. Cell 79: 353–364. 7954801 10.1016/0092-8674(94)90203-8

[2] Bouchier-Hayes L, Lartigue L, Newmeyer DD (2005) Mitochondria: pharmacological manipulation of cell death. J Clin Invest 115: 2640–2647. 16200197 10.1172/JCI26274 PMC1236694

[3] Newmeyer DD, Ferguson-Miller S (2003) Mitochondria. Releasing power for life and unleashing the machineries of death. Cell 112: 481–490. 12600312 10.1016/s0092-8674(03)00116-8

[4] Tait SW, Parsons MJ, Llambi F, Bouchier-Hayes L, Connell S, et al (2010) Resistance to caspase-independent cell death requires persistence of intact mitochondria. Dev Cell 18: 802–813. 20493813 10.1016/j.devcel.2010.03.014 PMC3004027

[5] Antonsson B, Montessuit S, Sanchez B, Martinou JC (2001) Bax is present as a high molecular weight oligomer/complex in the mitochondrial membrane of apoptotic cells. J Biol Chem 276: 11615–11623. 11136736 10.1074/jbc.M010810200

[6] Wolter KG, Hsu YT, Smith CL, Nechushtan A, Xi XG, et al (1997) Movement of Bax from the cytosol to mitochondria during apoptosis. J Cell Biol 139: 1281–1292. 9382873 10.1083/jcb.139.5.1281 PMC2140220

[7] Hsu YT, Youle RJ (1997) Nonionic detergents induce dimerization among members of the Bcl-2 family. J Biol Chem 272: 13829–13834. 9153240 10.1074/jbc.272.21.13829

[8] Hsu YT, Wolter KG, Youle RJ (1997) Cytosol-to-membrane redistribution of Bax and Bcl-X(L) during apoptosis. Proc Natl Acad Sci U S A 94: 3668–3672. 9108035 10.1073/pnas.94.8.3668 PMC20498

[9] Kuwana T, Mackey MR, Perkins G, Ellisman MH, Latterich M, et al (2002) Bid, Bax, and lipids cooperate to form supramolecular openings in the outer mitochondrial membrane. Cell 111: 331–342. 12419244 10.1016/s0092-8674(02)01036-x

[10] Wei MC, Lindsten T, Mootha VK, Weiler S, Gross A, et al (2000) tBID, a membrane-targeted death ligand, oligomerizes BAK to release cytochrome c. Genes Dev 14: 2060–2071. 10950869 PMC316859

[11] Wei MC, Zong WX, Cheng EH, Lindsten T, Panoutsakopoulou V, et al (2001) Proapoptotic BAX and BAK: a requisite gateway to mitochondrial dysfunction and death. Science 292: 727–730. 11326099 10.1126/science.1059108 PMC3049805

[12] Griffiths GJ, Dubrez L, Morgan CP, Jones NA, Whitehouse J, et al (1999) Cell damage-induced conformational changes of the pro-apoptotic protein Bak in vivo precede the onset of apoptosis. J Cell Biol 144: 903–914. 10085290 10.1083/jcb.144.5.903 PMC2148192

[13] Slee EA, Harte MT, Kluck RM, Wolf BB, Casiano CA, et al (1999) Ordering the cytochrome c-initiated caspase cascade: hierarchical activation of caspases-2, -3, -6, -7, -8, and -10 in a caspase-9-dependent manner. J Cell Biol 144: 281–292. 9922454 10.1083/jcb.144.2.281 PMC2132895

[14] Acehan D, Jiang X, Morgan DG, Heuser JE, Wang X, et al (2002) Three-dimensional structure of the apoptosome: implications for assembly, procaspase-9 binding, and activation. Mol Cell 9: 423–432. 11864614 10.1016/s1097-2765(02)00442-2

[15] Lavrik IN, Golks A, Krammer PH (2005) Caspases: pharmacological manipulation of cell death. J Clin Invest 115: 2665–2672. 16200200 10.1172/JCI26252 PMC1236692

[16] Liu X, Kim CN, Yang J, Jemmerson R, Wang X (1996) Induction of apoptotic program in cell-free extracts: requirement for dATP and cytochrome c. Cell 86: 147–157. 8689682 10.1016/s0092-8674(00)80085-9

[17] Nicholson DW (1999) Caspase structure, proteolytic substrates, and function during apoptotic cell death. Cell Death Differ 6: 1028–1042. 10578171 10.1038/sj.cdd.4400598

[18] Taylor RC, Brumatti G, Ito S, Hengartner MO, Derry WB, et al (2007) Establishing a blueprint for CED-3-dependent killing through identification of multiple substrates for this protease. J Biol Chem 282: 15011–15021. 17371877 10.1074/jbc.M611051200

[19] Lartigue L, Kushnareva Y, Seong Y, Lin H, Faustin B, et al (2009) Caspase-independent mitochondrial cell death results from loss of respiration, not cytotoxic protein release. Mol Biol Cell 20: 4871–4884. 19793916 10.1091/mbc.E09-07-0649 PMC2785731

[20] Colell A, Ricci JE, Tait S, Milasta S, Maurer U, et al (2007) GAPDH and autophagy preserve survival after apoptotic cytochrome c release in the absence of caspase activation. Cell 129: 983–997. 17540177 10.1016/j.cell.2007.03.045

[21] Kuwana T, Bouchier-Hayes L, Chipuk JE, Bonzon C, Sullivan BA, et al (2005) BH3 domains of BH3-only proteins differentially regulate Bax-mediated mitochondrial membrane permeabilization both directly and indirectly. Mol Cell 17: 525–535. 15721256 10.1016/j.molcel.2005.02.003

[22] Estaquier J, Arnoult D (2007) Inhibiting Drp1-mediated mitochondrial fission selectively prevents the release of cytochrome c during apoptosis. Cell Death Differ 14: 1086–1094. 17332775 10.1038/sj.cdd.4402107

[23] Frezza C, Cipolat S, Martins de Brito O, Micaroni M, Beznoussenko GV, et al (2006) OPA1 controls apoptotic cristae remodeling independently from mitochondrial fusion. Cell 126: 177–189. 16839885 10.1016/j.cell.2006.06.025

[24] Scorrano L, Ashiya M, Buttle K, Weiler S, Oakes SA, et al (2002) A distinct pathway remodels mitochondrial cristae and mobilizes cytochrome c during apoptosis. Dev Cell 2: 55–67. 11782314 10.1016/s1534-5807(01)00116-2

[25] Yamaguchi R, Lartigue L, Perkins G, Scott RT, Dixit A, et al (2008) Opa1-mediated cristae opening is Bax/Bak and BH3 dependent, required for apoptosis, and independent of Bak oligomerization. Mol Cell 31: 557–569. 18691924 10.1016/j.molcel.2008.07.010 PMC2636708

[26] Lovell JF, Billen LP, Bindner S, Shamas-Din A, Fradin C, et al (2008) Membrane binding by tBid initiates an ordered series of events culminating in membrane permeabilization by Bax. Cell 135: 1074–1084. 19062087 10.1016/j.cell.2008.11.010

[27] Terrones O, Antonsson B, Yamaguchi H, Wang HG, Liu J, et al (2004) Lipidic pore formation by the concerted action of proapoptotic BAX and tBID. J Biol Chem 279: 30081–30091. 15138279 10.1074/jbc.M313420200

[28] Schafer B, Quispe J, Choudhary V, Chipuk JE, Ajero TG, et al (2009) Mitochondrial outer membrane proteins assist Bid in Bax-mediated lipidic pore formation. Mol Biol Cell 20: 2276–2285. 19244344 10.1091/mbc.E08-10-1056 PMC2669034

[29] Zaltsman Y, Shachnai L, Yivgi-Ohana N, Schwarz M, Maryanovich M, et al (2010) MTCH2/MIMP is a major facilitator of tBID recruitment to mitochondria. Nat Cell Biol 12: 553–562. 20436477 10.1038/ncb2057 PMC4070879

[30] Montessuit S, Somasekharan SP, Terrones O, Lucken-Ardjomande S, Herzig S, et al (2010) Membrane remodeling induced by the dynamin-related protein Drp1 stimulates Bax oligomerization. Cell 142: 889–901. 20850011 10.1016/j.cell.2010.08.017 PMC4115189

[31] Cassidy-Stone A, Chipuk JE, Ingerman E, Song C, Yoo C, et al (2008) Chemical inhibition of the mitochondrial division dynamin reveals its role in Bax/Bak-dependent mitochondrial outer membrane permeabilization. Dev Cell 14: 193–204. 18267088 10.1016/j.devcel.2007.11.019 PMC2267902

[32] Brustovetsky N, Dubinsky JM, Antonsson B, Jemmerson R (2003) Two pathways for tBID-induced cytochrome c release from rat brain mitochondria: BAK- versus BAX-dependence. J Neurochem 84: 196–207. 12485416 10.1046/j.1471-4159.2003.01545.x

[33] Terrones O, Etxebarria A, Landajuela A, Landeta O, Antonsson B, et al (2008) BIM and tBID are not mechanistically equivalent when assisting BAX to permeabilize bilayer membranes. J Biol Chem 283: 7790–7803. 18195012 10.1074/jbc.M708814200

[34] Polster BM, Kinnally KW, Fiskum G (2001) BH3 death domain peptide induces cell type-selective mitochondrial outer membrane permeability. J Biol Chem 276: 37887–37894. 11483608 10.1074/jbc.M104552200

[35] Cheng EH, Sheiko TV, Fisher JK, Craigen WJ, Korsmeyer SJ (2003) VDAC2 inhibits BAK activation and mitochondrial apoptosis. Science 301: 513–517. 12881569 10.1126/science.1083995

[36] Csordas G, Renken C, Varnai P, Walter L, Weaver D, et al (2006) Structural and functional features and significance of the physical linkage between ER and mitochondria. J Cell Biol 174: 915–921. 16982799 10.1083/jcb.200604016 PMC2064383

[37] Billen LP, Kokoski CL, Lovell JF, Leber B, Andrews DW (2008) Bcl-XL inhibits membrane permeabilization by competing with Bax. PLoS Biol 6: e147 doi:10.1371/journal.pbio.0060147. 18547146 10.1371/journal.pbio.0060147 PMC2422857

[38] Epand RF, Martinou JC, Montessuit S, Epand RM (2002) Membrane perturbations induced by the apoptotic Bax protein. Biochem J 367: 849–855. 12180909 10.1042/BJ20020986 PMC1222950

[39] Saito M, Korsmeyer SJ, Schlesinger PH (2000) BAX-dependent transport of cytochrome c reconstituted in pure liposomes. Nat Cell Biol 2: 553–555. 10934477 10.1038/35019596

[40] Kluck RM, Esposti MD, Perkins G, Renken C, Kuwana T, et al (1999) The pro-apoptotic proteins, Bid and Bax, cause a limited permeabilization of the mitochondrial outer membrane that is enhanced by cytosol. J Cell Biol 147: 809–822. 10562282 10.1083/jcb.147.4.809 PMC2156156

[41] Wojtczak L, Zaluska H, Wroniszewska A, Wojtczak AB (1972) Assay for the intactness of the outer membrane in isolated mitochondria. Acta Biochim Pol 19: 227–234. 4347175

[42] Kushnareva Y, Newmeyer DD (2010) Bioenergetics and cell death. Ann N Y Acad Sci 1201: 50–57. 20649539 10.1111/j.1749-6632.2010.05633.x PMC3079367

[43] Lemeshko VV (2002) Cytochrome c sorption-desorption effects on the external NADH oxidation by mitochondria: experimental and computational study. J Biol Chem 277: 17751–17757. 11886867 10.1074/jbc.M201002200

[44] Oltersdorf T, Elmore SW, Shoemaker AR, Armstrong RC, Augeri DJ, et al (2005) An inhibitor of Bcl-2 family proteins induces regression of solid tumours. Nature 435: 677–681. 15902208 10.1038/nature03579

[45] Du H, Wolf J, Schafer B, Moldoveanu T, Chipuk JE, et al (2011) BH3 domains other than Bim and Bid can directly activate Bax/Bak. J Biol Chem 286: 491–501. 21041309 10.1074/jbc.M110.167148 PMC3013008

[46] Edlich F, Banerjee S, Suzuki M, Cleland MM, Arnoult D, et al (2011) Bcl-x(L) retrotranslocates Bax from the mitochondria into the cytosol. Cell 145: 104–116. 21458670 10.1016/j.cell.2011.02.034 PMC3070914

[47] Kuwana T, Bouchier-Hayes L, Chipuk JE, Bonzon C, Sullivan BA, et al (2005) BH3 domains of BH3-only proteins differentially regulate Bax-mediated mitochondrial membrane permeabilization both directly and indirectly. Mol Cell 17: 525–535. 15721256 10.1016/j.molcel.2005.02.003

[48] Leber B, Lin J, Andrews DW (2007) Embedded together: the life and death consequences of interaction of the Bcl-2 family with membranes. Apoptosis 12: 897–911. 17453159 10.1007/s10495-007-0746-4 PMC2868339

[49] Llambi F, Moldoveanu T, Tait SW, Bouchier-Hayes L, Temirov J, et al (2011) A unified model of mammalian BCL-2 protein family interactions at the mitochondria. Mol Cell 44: 517–531. 22036586 10.1016/j.molcel.2011.10.001 PMC3221787

[50] Walensky LD, Pitter K, Morash J, Oh KJ, Barbuto S, et al (2006) A stapled BID BH3 helix directly binds and activates BAX. Mol Cell 24: 199–210. 17052454 10.1016/j.molcel.2006.08.020

[51] Willis SN, Fletcher JI, Kaufmann T, van Delft MF, Chen L, et al (2007) Apoptosis initiated when BH3 ligands engage multiple Bcl-2 homologs, not Bax or Bak. Science 315: 856–859. 17289999 10.1126/science.1133289

[52] Perez D, White E (2000) TNF-alpha signals apoptosis through a bid-dependent conformational change in Bax that is inhibited by E1B 19K. Mol Cell 6: 53–63. 10949027

[53] Eskes R, Desagher S, Antonsson B, Martinou JC (2000) Bid induces the oligomerization and insertion of Bax into the outer mitochondrial membrane. Mol Cell Biol 20: 929–935. 10629050 10.1128/mcb.20.3.929-935.2000 PMC85210

[54] Bleicken S, Classen M, Padmavathi PV, Ishikawa T, Zeth K, et al (2010) Molecular details of Bax activation, oligomerization, and membrane insertion. J Biol Chem 285: 6636–6647. 20008353 10.1074/jbc.M109.081539 PMC2825459

[55] Ren D, Tu HC, Kim H, Wang GX, Bean GR, et al (2010) BID, BIM, and PUMA are essential for activation of the BAX- and BAK-dependent cell death program. Science 330: 1390–1393. 21127253 10.1126/science.1190217 PMC3163443

[56] Ott M, Norberg E, Walter KM, Schreiner P, Kemper C, et al (2007) The mitochondrial TOM complex is required for tBid/Bax-induced cytochrome c release. J Biol Chem 282: 27633–27639. 17635912 10.1074/jbc.M703155200

[57] Bellot G, Cartron PF, Er E, Oliver L, Juin P, et al (2007) TOM22, a core component of the mitochondria outer membrane protein translocation pore, is a mitochondrial receptor for the proapoptotic protein Bax. Cell Death Differ 14: 785–794. 17096026 10.1038/sj.cdd.4402055

[58] Etxebarria A, Terrones O, Yamaguchi H, Landajuela A, Landeta O, et al (2009) Endophilin B1/Bif-1 stimulates BAX activation independently from its capacity to produce large scale membrane morphological rearrangements. J Biol Chem 284: 4200–4212. 19074440 10.1074/jbc.M808050200 PMC3837389

[59] Roucou X, Montessuit S, Antonsson B, Martinou JC (2002) Bax oligomerization in mitochondrial membranes requires tBid (caspase-8-cleaved Bid) and a mitochondrial protein. Biochem J 368: 915–921. 12193163 10.1042/BJ20020972 PMC1223025

[60] Lepock JR, Frey HE, Ritchie KP (1993) Protein denaturation in intact hepatocytes and isolated cellular organelles during heat shock. J Cell Biol 122: 1267–1276. 8376462 10.1083/jcb.122.6.1267 PMC2119851

[61] Pagliari LJ, Kuwana T, Bonzon C, Newmeyer DD, Tu S, et al (2005) The multidomain proapoptotic molecules Bax and Bak are directly activated by heat. Proc Natl Acad Sci U S A 102: 17975–17980. 16330765 10.1073/pnas.0506712102 PMC1312392

[62] Parone PA, James DI, Da Cruz S, Mattenberger Y, Donze O, et al (2006) Inhibiting the mitochondrial fission machinery does not prevent Bax/Bak-dependent apoptosis. Mol Cell Biol 26: 7397–7408. 17015472 10.1128/MCB.02282-05 PMC1636857

[63] Sheridan C, Martin SJ (2010) Mitochondrial fission/fusion dynamics and apoptosis. Mitochondrion 10: 640–648. 20727425 10.1016/j.mito.2010.08.005

[64] Sheridan C, Delivani P, Cullen SP, Martin SJ (2008) Bax- or Bak-induced mitochondrial fission can be uncoupled from cytochrome C release. Mol Cell 31: 570–585. 18722181 10.1016/j.molcel.2008.08.002

[65] Lee YJ, Jeong SY, Karbowski M, Smith CL, Youle RJ (2004) Roles of the mammalian mitochondrial fission and fusion mediators Fis1, Drp1, and Opa1 in apoptosis. Mol Biol Cell 15: 5001–5011. 15356267 10.1091/mbc.E04-04-0294 PMC524759

[66] Costa V, Giacomello M, Hudec R, Lopreiato R, Ermak G, et al (2010) Mitochondrial fission and cristae disruption increase the response of cell models of Huntington's disease to apoptotic stimuli. EMBO Mol Med 2: 490–503. 21069748 10.1002/emmm.201000102 PMC3044888

[67] Chang CR, Manlandro CM, Arnoult D, Stadler J, Posey AE, et al (2010) A lethal de novo mutation in the middle domain of the dynamin-related GTPase Drp1 impairs higher order assembly and mitochondrial division. J Biol Chem 285: 32494–32503. 20696759 10.1074/jbc.M110.142430 PMC2952251

[68] Smirnova E, Griparic L, Shurland DL, van der Bliek AM (2001) Dynamin-related protein Drp1 is required for mitochondrial division in mammalian cells. Mol Biol Cell 12: 2245–2256. 11514614 10.1091/mbc.12.8.2245 PMC58592

[69] Zhou HX (2009) Crowding effects of membrane proteins. J Phys Chem B 113: 7995–8005. 19323472 10.1021/jp8107446 PMC2742981

[70] Martinez-Caballero S, Dejean LM, Kinnally MS, Oh KJ, Mannella CA, et al (2009) Assembly of the mitochondrial apoptosis-induced channel, MAC. J Biol Chem 284: 12235–12245. 19261612 10.1074/jbc.M806610200 PMC2673292

[71] Lin SH, Perera MN, Nguyen T, Datskovskiy D, Miles M, et al (2011) Bax forms two types of channels, one of which is voltage-gated. Biophys J 101: 2163–2169. 22067154 10.1016/j.bpj.2011.09.041 PMC3207152

[72] Qian S, Wang W, Yang L, Huang HW (2008) Structure of transmembrane pore induced by Bax-derived peptide: evidence for lipidic pores. Proc Natl Acad Sci U S A 105: 17379–17383. 18987313 10.1073/pnas.0807764105 PMC2582298

[73] Garcia-Saez AJ, Coraiola M, Dalla Serra M, Mingarro I, Menestrina G, et al (2005) Peptides derived from apoptotic Bax and Bid reproduce the poration activity of the parent full-length proteins. Biophys J 88: 3976–3990. 15778450 10.1529/biophysj.104.058008 PMC1305629

[74] Basanez G, Nechushtan A, Drozhinin O, Chanturiya A, Choe E, et al (1999) Bax, but not Bcl-xL, decreases the lifetime of planar phospholipid bilayer membranes at subnanomolar concentrations. Proc Natl Acad Sci U S A 96: 5492–5497. 10318911 10.1073/pnas.96.10.5492 PMC21887

[75] Zhou L, Chang DC (2008) Dynamics and structure of the Bax-Bak complex responsible for releasing mitochondrial proteins during apoptosis. J Cell Sci 121: 2186–2196. 18544634 10.1242/jcs.024703

[76] Lucken-Ardjomande S, Martinou JC (2005) Newcomers in the process of mitochondrial permeabilization. J Cell Sci 118: 473–483. 15673686 10.1242/jcs.01654

[77] McMahon HT, Kozlov MM, Martens S (2010) Membrane curvature in synaptic vesicle fusion and beyond. Cell 140: 601–605. 20211126 10.1016/j.cell.2010.02.017

[78] Campelo F, Fabrikant G, McMahon HT, Kozlov MM (2010) Modeling membrane shaping by proteins: focus on EHD2 and N-BAR domains. FEBS Lett 584: 1830–1839. 19836393 10.1016/j.febslet.2009.10.023

[79] Rostovtseva TK, Boukari H, Antignani A, Shiu B, Banerjee S, et al (2009) Bax activates endophilin B1 oligomerization and lipid membrane vesiculation. J Biol Chem 284: 34390–34399. 19805544 10.1074/jbc.M109.021873 PMC2797207

[80] Lapidus RG, Sokolove PM (1993) Spermine inhibition of the permeability transition of isolated rat liver mitochondria: an investigation of mechanism. Arch Biochem Biophys 306: 246–253. 8215411 10.1006/abbi.1993.1507

[81] Andreyev AY, Shen Z, Guan Z, Ryan A, Fahy E, et al (2009) Application of proteomic marker ensembles to subcellular organelle identification. Mol Cell Proteomics 10.1074/mcp.M900432-MCP200 PMC2830848 19884172

[82] von Ahsen O, Renken C, Perkins G, Kluck RM, Bossy-Wetzel E, et al (2000) Preservation of mitochondrial structure and function after Bid- or Bax-mediated cytochrome c release. J Cell Biol 150: 1027–1036. 10973993 10.1083/jcb.150.5.1027 PMC2175243

[83] Suzuki M, Youle RJ, Tjandra N (2000) Structure of Bax: coregulation of dimer formation and intracellular localization. Cell 103: 645–654. 11106734 10.1016/s0092-8674(00)00167-7

[84] Luciano F, Jacquel A, Colosetti P, Herrant M, Cagnol S, et al (2003) Phosphorylation of Bim-EL by Erk1/2 on serine 69 promotes its degradation via the proteasome pathway and regulates its proapoptotic function. Oncogene 22: 6785–6793. 14555991 10.1038/sj.onc.1206792

[85] Ivanova PT, Milne SB, Byrne MO, Xiang Y, Brown HA (2007) Glycerophospholipid identification and quantitation by electrospray ionization mass spectrometry. Methods Enzymol 432: 21–57. 17954212 10.1016/S0076-6879(07)32002-8

[86] Ricchelli F, Gobbo S, Moreno G, Salet C (1999) Changes of the fluidity of mitochondrial membranes induced by the permeability transition. Biochemistry 38: 9295–9300. 10413503 10.1021/bi9900828

[87] Arrigoni O, Singer TP (1962) Limitations of the phenazine methosulphate assay for succinic and related dehydrogenases. Nature 193: 1256–1258. 13862582 10.1038/1931256a0

